# Comprehensive survey of radionuclides in contemporary smokeless tobacco products

**DOI:** 10.1186/s13065-017-0359-0

**Published:** 2017-12-19

**Authors:** K. McAdam, H. Kimpton, A. Porter, C. Liu, A. Faizi, M. Mola, J. McAughey, B. Rodu

**Affiliations:** 1 Group Research & Development, British American Tobacco, Regents Park Road, Southampton, SO15 8TL UK; 2 3810 St. Antoine W, Montreal, QC H4C 1B4 Canada; 3 Department of Medicine, School of Medicine, University of Louisville, 505 South Hancock Street, Louisville, KY 40202 USA

**Keywords:** Smokeless tobacco, Snuff, Snus, Radionuclides, Radioactivity, Potassium-40

## Abstract

There is considerable interest in the chemical composition of smokeless tobacco products (STPs), owing to health concerns associated with their use. Previous studies have documented levels of ^210^Po, ^210^Pb and uranium in STP samples. Here, the levels of 13 α-particle and 15 β-radiation emitting radionuclides have been measured in a broad and representative range of contemporary STPs commercially available in the United States and Sweden. For each radionuclide, the level of radioactivity and calculated mass per gram of STP are reported. The results indicate that, among 34 Swedish snus and 44 US STPs, a more complex radionuclide content exists than previously reported for these products. Of the 28 radionuclides examined, 13 were detected and quantified in one or more STPs. The most frequently identified radionuclides in these STPs were ^40^K, ^14^C, ^210^Po and ^226^Ra. Over half the STPs also contained ^228^Th, and an additional 8 radionuclides were identified in a small number of STPs. The presence of ^14^C, ^3^H and ^230^Th are reported in tobacco for the first time. The activity of β-emitters was much greater than those of α-emitters, and the β-emitter ^40^K was present in the STPs with both the greatest radioactivity and mass concentrations. Since the three radionuclides included in the FDA’s HPHC list were either not detected (^235^U), identified in only three of 78 samples (^238^U), and/or had activity levels over fifty times lower than that of ^40^K (^210^Po, ^238^U), there may be a rationale for reconsidering the radionuclides currently included in the FDA HPHC list, particularly with respect to ^40^K. Using a model of the physical and biological compartments which must be considered to estimate the exposure of STP users to radionuclides, we conclude that exposure from α-emitters may be minimal to STP users, but ^40^K in particular may expose the oral cavities of STP users to β-radiation. Although a more comprehensive picture of the radioisotope content of STPs has emerged from this study, epidemiological evidence suggests that the levels of radionuclides measured in this study appear unlikely to present significant risks to STP users.

**Electronic supplementary material:**

The online version of this article (10.1186/s13065-017-0359-0) contains supplementary material, which is available to authorized users.

## Introduction

There has been considerable interest in recent years in the chemical composition of smokeless tobacco products (STPs), primarily based around health concerns associated with their use. Although banned in the European Union, STPs are widely used in the United States, Sweden and Norway, and across large parts of Africa and Asia. The International Agency for Research on Cancer (IARC) has classified STPs collectively as Group 1 (known human carcinogens) [1]. However, worldwide there are very different types of STP used [1], including dry snuff (DS), moist snuff (MS), chewing tobacco (CT), hard pellets (HP) and soft pellets (SP) (predominantly in the USA), loose and pouched snus (predominately in Sweden), and a range of products used on the Indian sub-continent and in Africa. Indeed, a review of STPs by the UK Royal College of Physicians noted that different health risks are associated with the use of different STPs in line with the levels of chemical toxicants within those products [2]. In an examination of the risks associated with use of STPs [1], *IARC Monograph 89* identified 28 chemical agents or toxicants that have been reported in STPs, including the radioactive elements polonium (^210^Po) in US STPs [3] and uranium in Indian STPs [4], with the latter cited in IARC Monograph 89 as uranium-235 (^235^U) and uranium-238 (^238^U) [1]. These radionuclides have subsequently been identified by the FDA as “Harmful or Potentially Harmful Constituents” (HPHC) in tobacco products and tobacco smoke [5]. A recent revision to IARC’s consideration of STPs revised the summary list to ^210^Po and uranium [6].

The radioactive content of tobacco, cigarette smoke and ash has been the focus of research since the early 1950s [7]. Since then a wide range of radionuclides have been identified in tobacco [8]. The 2008 report from the Scientific Committee on Emerging and Newly Identified Health Risks (SCENIHR) recognised that the radionuclide content of tobacco used for STP manufacture was important in determining the radionuclide content of STPs, and stated that radium-226 (^226^Ra), and to some extent lead-210 (^210^Pb), a progeny of ^226^Ra, were the most important radionuclides in the tobaccos used to manufacture STPs [9]. SCENIHR also concluded that “the dose of ionising radiation from these sources must be considered as negligible in comparison e.g. with the natural radiation background and other sources of ionising radiation”. Based on previous studies of the radionuclide content of tobacco and other plant materials, it seems likely that many more radionuclides are present in STPs in addition to the five listed by IARC and SCENIHR [1, 9].

The main types of radionuclides that have been identified in plants arise from four distinct sources [10], three natural and one anthropogenic. The first group consists of primordial radionuclides incorporated into the planet during its formation, with half-lives comparable to the age of the earth. These include potassium-40 (^40^K), thorium-232 (^232^Th) and uranium-238 (^238^U). The second group comprises the decay products or progeny of the primordial elements, which are collected into radionuclide groups known as decay series, including the ^238^U series, ^232^Th series and actinium series of radionuclides. The half-lives of these radionuclides cover many orders of magnitude, from thousands of years to fractions of seconds, and include ^210^Pb, ^210^Po and ^226^Ra. The third group includes radioactive isotopes continuously produced in the earth’s atmosphere by cosmic ray bombardment, such as the β-emitters: tritium (^3^H), carbon-14 (^14^C), and phosphorous-32 (^32^P). The final group comprises man-made radionuclides arising in the environment principally from nuclear weapons testing and the nuclear power industry, as well as contributions from specialised (e.g. medical) uses. Examples of this group include caesium-137 (^137^Cs), iodine-131 (^131^I), strontium-90 (^90^Sr) and plutonium radionuclides [11].

Environmental radionuclides enter the human body due to their ubiquitous presence in food, water, and air. Use of products containing tobacco can act as an additional exposure source since radionuclides may be present in tobacco, as in all plants, through uptake of compounds from soil, direct deposition onto leaves, or incorporation of atmospheric gases into the growing plant.

IARC has classified as carcinogenic to humans (Group 1) all radionuclides internalized within the human body that emit α-particles or β-particles for the following reasons. First, all α-particles emitted by radionuclides, irrespective of their source, produce the same pattern of secondary ionizations and the same pattern of localized damage to biological molecules, including DNA. These effects, most easily studied in vitro, include DNA double-strand breaks, chromosomal aberrations, gene mutations and cell transformation. The same is true for all β-particles. Second, all radionuclides that emit α-particles and that have been adequately studied have been shown to cause cancer in humans and in experimental animals. The same is true for β-particles including ^3^H, which produces β-particles of very low energy, but for which there is nonetheless *sufficient evidence* of carcinogenicity in experimental animals. Third, α-particles emitted by radionuclides, irrespective of their source, have been shown to cause chromosomal aberrations in circulating lymphocytes and gene mutations in humans in vivo. Again, the same is true for β-particles: the evidence from studies in humans and experimental animals suggests that similar doses to the same tissues—for example, lung cells or bone surfaces—from β-particles emitted during the decay of different radionuclides produce the same types of non-neoplastic effects and cancers. IARC has recently also established that there is sufficient evidence in humans for the carcinogenicity of γ-radiation, and has assigned this form of radiation to Group 1, along with α- and β-emitters [12]. In addition, IARC has identified specific radionuclides as Group 1 carcinogens. There is evidence of carcinogenicity in humans for ^226^Ra, ^224^Ra, and ^228^Ra; ^232^Th and its decay products; plutonium-239 (with plutonium-240; ^239,240^Pu); phosphorus-32 (^32^P); and ^131^I [11]. There is evidence of carcinogenicity in animals for many more radionuclides [12].

Given the current scientific and regulatory focus on toxicants in STPs, the paucity of studies investigating the presence of radionuclides in STPs, in comparison to the wider range of radionuclides identified in tobacco [8], highlights a significant need for thorough investigation of STP radionuclide contents. The aim of the present study therefore was to identify the levels of radionuclides in a comprehensive range of contemporary STPs, representing seven different types of product [13–15]. In total, 78 products representing approximately 90% of the market share for the major categories of STP in the United States and Sweden [13] were analyzed by alpha spectrometry, liquid scintillation counting and gamma spectrometry for the activity and concentration of 13 α-particle and 15 β-radiation emitters (Table 1) broadly representative of the four major sources of radioactivity found in the environment.

**Table 1:** Radionuclides examined in the current study

Radionuclide	Symbol	Main source	Main radioactive emission mode	How measured (current study)	Half-life time	Specific activity
Uranium-235	^235^U	Actinium series	α	A	7.04 × 10^8^ years	79.8 kBq/g
Uranium-238	^238^U	Primordial	α	A	4.47 × 10^9^ years	12.44 kBq/g
Uranium-234	^234^U	Uranium-238 decay series	α	A	2.455 × 10^5^ years	231.3 MBq/g
Thorium-234	^234^Th		β^−^	G	24.1 days	860 TBq/g
Protactinium-234	^234m^Pa		β^−^	G	6.7 h	74,000 TBq/g
Thorium-230	^230^Th		α	A	75,440 years	762.8 MBq/g
Radium-226	^226^Ra		α	A	1599 years	36 GBq/g
Lead-214	^214^Pb		β^−^	G	26.9 min	1.213 × 10^6^ TBq/g
Bismuth-214	^214^Bi		α	G	28.7 min	1.63 × 10^6^ TBq/g
Lead-210	^210^Pb		β^−^	G	22.6 years	2.84 TBq/g
Polonium-210	^210^Po		α	A	138.4 days	166.272 TBq/g
Thorium-232	^232^Th	Primordial	α	A	1.4 × 10^10^ years	4.07 kBq/g
Actinium-228	^228^Ac	Thorium-232 decay series	β^−^	G	6.15 h	82,800 TBq/g
Thorium-228	^228^Th		α	A	1.913 years	30.366 TBq/g
Lead-212	^212^Pb		β^−^	G	10.6 h	51,407 TBq/g
Bismuth-212	^212^Bi		β^−^	G	1.009 h	542,000 TBq/g
Tantalum-208	^208^Tl		β^−^	G	183.2 s	1.096 × 10^7^ TBq/g
Potassium-40	^40^K	Primordial	β^−^	G	1.248 × 10^9^ years	265.4 kBq/g
Carbon-14	^14^C	Cosmic ray	β^−^	G	5700 years	170 GBq/g
Tritium	^3^H		β^−^	G	12.32 years	356.2 TBq/g
Americium-241	^241^Am	Anthropogenic	α	G	432.5 years	126.9 GBq/g
Plutonium-238	^238^Pu		α	G	87.8 years	634 GBq/g
Plutonium-239	^239^Pu		α	A	24,110 years	2.297 GBq/g
Plutonium-240	^240^Pu		α	A	6561 years	8.404 GBq/g
Cesium-137	^137^Cs		β^−^	G	30.08 years	3.214 TBq/g
Cesium-134	^134^Cs		β^−^	G	2.0652 years	47.864 TBq/g
Iodine-131	^131^I		β^−^	G	8.0252 days	4598.8 TBq/g
Cobalt-60	^60^Co		β^−^	G	5.2749 years	41.868 TBq/g

*A* α-spectroscopy, *G* γ-spectrometry

## Experimental section

### Tobacco samples

The survey was conducted in two parts, with an initial sampling of 70 STPs from the United States and Sweden in 2008 [13], and a second sampling of 73 STPs in 2010 [14], conducted in order to ensure that the ages of the samples at the time of analysis were reflective of consumption patterns.

Details of the STP markets in the United States and Sweden were obtained in 2008, and the products for analysis were chosen to reflect approximately 90% share of these two markets at that time, including STPs from all of the principal manufacturers. A similar approach was adopted in 2010, when more than 90% of the first set of STPs were resampled, but some samples were no longer on-sale. Eight new products were sampled. These included replacements for the products no-longer sold, and examples of a new category of STP (US snus) that was not available during the 2008 exercise. In total 78 different STPs were sampled.

Both samplings included the major products in each category of STP; where there were multiple flavored variants, the base product was sampled and analyzed. In total, the survey comprised 34 Swedish products (10 L snus and 24 P snus) and 44 US products (13 CT, 5 DS, 2 HP, 1 SP, 16 MS, 6 US snus and 1 plug product) (Additional file 1: Table S1).

In both sampling exercises the products were sourced from Swedish retail websites or from retail outlets in the USA, imported into the United Kingdom, and kept frozen at − 20 °C until analysis.

### Reagents

All laboratory reagents (hydrochloric acid, hydrofluoric acid, nitric acid, sulfuric acid, ferric hydroxide, copper oxide, TEA and EDTA) were from Thermo Fisher Scientific Inc. and were of Analytical Reagent Grade.

Barium-133 internal tracer was supplied by Amersham International. Polonium-208, thorium-229 and plutonium-242 internal tracers were supplied by the National Physical Laboratory (UK). Uranium-232 internal tracer was supplied by Harwell Technology (Oxford, UK).

### Measurement of water content in the STP samples

To convert measurements made on a wet-weight basis (wwb) to a dry-weight basis (dwb), the water content of all STPs was measured by near-infrared (NIR) spectroscopy using a standard technique wherein water was extracted from the STPs using dry methanol. A calibrated double-beam spectrometer was used to measure the intensity of the combination band at 1943 nm (due to –OH stretching and H–OH bending of the water molecule); intensities were compared to standards containing water in methanol for the purposes of quantification.

### Measurement of ash content of STPs

The inorganic material content of STPs was estimated by heating the STP at 500–550 °C in air in a pre-dried silica dish placed in a muffle furnace for 1 h. Organic material present in the sample during this time period was burnt off as combustion gases; if the resulting ash was not uniformly white (the presence of carbon particles in the ash indicates incomplete ashing of the STP) then the samples were ashed for a further 30 min. The residual sample weight after ashing, with allowance for the original moisture content of the STP, provided an estimate of the STP’s inorganic content.

### Determination of radionuclides

The radionuclides examined in the present study are listed in Table 1. Also summarized in the table are their sources, main radioactive decay modes, measurement methods in this study, half-lives and specific activities. All radionuclide analyses were conducted by Environmental Scientifics Group (Didcot, UK), from whom further method details can be obtained.

#### ^210^Po

^210^Po was determined by wet oxidation. ^208^Po was added to the sample as an internal tracer. A nitric acid/hydrofluoric acid mixture was added to an aliquot of the homogenized sample and then taken to dryness. This was repeated, then nitric acid was added and the sample taken to dryness to remove any traces of hydrofluoric acid. The residue was dissolved in hydrochloric acid, and polonium was isolated by auto deposition onto a silver disc (Fourjay Limited, UK) under reducing conditions. The radioactivity on the silver disc was measured by alpha spectrometry to determine the ratio of ^210^Po to ^208^Po.

#### ^226^Ra

Levels of ^226^Ra were determined by adding a known activity of ^133^Ba tracer to a dried and ground aliquot of the sample, which was then ashed in a furnace overnight. The sample was then digested in aqua regia (3:1 mix of hydrochloric:nitric acid). The radium radionuclides were initially co-precipitated with lead and barium sulfates from a faintly acidic water sample. The precipitate was isolated by centrifuging, then redissolved in an alkaline solution of ethylenediaminetetraacetic acid (EDTA) and triethanolamine (TEA). The radium radionuclides were then co-precipitated with barium sulphate from acetic acid medium free of lead contamination. The barium/radium sulphate was then further purified by a series of precipitations and finally mounted as a thin source on a 5 cm diameter stainless-steel planchet. Chemical recovery was determined by measurement of ^133^Ba by γ-ray spectrometry (High Purity Germanium Detector and NIM electronics, EG&G Ortec, AMETEK, Inc). After a 21-day ingrowth period, the source was counted for gross α-activity on a Berthold LB770 low-level proportional counter (LB 770 10-Channel α-β-low-level counter, Berthold Technologies GmbH & Co.) for 1000 min. This determines the α-activity of ^226^Ra and its daughters in secular equilibrium (^222^Rn, ^218^Po and ^214^Po). The ^226^Ra activity was given by dividing the gross α-activity by four.

#### Thorium isotopes (^232^Th, ^230^Th, ^228^Th)

An aliquot of the homogenized sample was spiked with a ^229^Th internal standard and then ashed at 450 °C. The ashed residue was dissolved in hydrofluoric acid. Thorium was concentrated by co-precipitation with ferric hydroxide. Following dissolution of the precipitate using nitric acid, the thorium was purified using ion-exchange chromatography (disposable plastic columns with Analytical Grade ion exchange resin, Eichrom Technologies, Inc.). The purified thorium was electrodeposited onto a stainless-steel disc (Fourjay Limited, UK), thorium activity was measured by α-spectrometry (Octéte, EG&G Ortec, AMETEK, Inc. and Alpha Analyst, Canberra UK Limited).

#### ^234^U, ^235^U and ^238^U

Uranium-232 internal yield tracer was added to a dried and ground aliquot of the sample and ashed in a furnace overnight. The ashed residue was dissolved in hydrochloric acid following pre-treatment with hydrofluoric and nitric acids. After co-precipitation of the uranium with ferric hydroxide, ion-exchange chromatography (disposable plastic columns with Analytical Grade ion exchange resin, Eichrom Technologies, Inc.) was used to further purify and separate the uranium, which was then electrodeposited onto stainless-steel discs (Fourjay Limited, UK). Measurement of the uranium isotopes was carried out by alpha-spectrometry.

#### ^238^Pu, ^239,240^Pu

Plutonium-242 yield tracer was added to a dried and ground aliquot of the sample and ashed in a furnace overnight. The sample was then digested in aqua regia. After co-precipitation of the nuclides of interest with ferric hydroxide, ion-exchange chromatography (disposable plastic columns with Analytical Grade ion exchange resin, Eichrom Technologies, Inc**)** was used to further purify and separate the plutonium from americium. The plutonium was then electrodeposited onto stainless-steel discs. Measurement of the plutonium isotopes was carried out by alpha-spectrometry.

#### ^3^H

A sub-sample of known weight was taken from each sample and then burnt in an oxygen rich atmosphere in the presence of a copper oxide catalyst. Under these conditions, the hydrogen species were converted to water vapor, which was then selectively trapped in a series of gas-bubblers containing 0.1 M nitric acid. Aliquots of known weight of this liquid were then assessed for their tritium content by liquid scintillation counting (1220 QUANTULUS Ultra Low Level Liquid Scintillation Spectrometer, PerkinElmer Inc.). The tritium activity was corrected for the proportion of the bubbler trapping solution taken and for the weight of sample combusted to yield the specific activity in the sample.

#### ^14^C

A sub-sample of known weight was taken from each sample and then burnt in an oxygen rich atmosphere in the presence of a copper oxide catalyst. Under these conditions, the carbon species were converted to carbon dioxide. This was then selectively trapped in a series of gas-bubblers containing a trapping medium. Aliquots of known weight were then assessed for their carbon-14 content by liquid scintillation counting (1220 QUANTULUS Ultra Low Level Liquid Scintillation Spectrometer, PerkinElmer Inc.). The carbon-14 activity was corrected for the proportion of the bubbler trapping solution taken and for the weight of sample combusted.

#### Gamma spectrometry

Gamma ray spectrometry was used to measure the activity of ^40^K, ^60^Co, ^131^I, ^134^Cs, ^137^Cs, ^208^Tl, ^210^Pb, ^212^Pb, ^212^Bi, ^214^Pb, ^214^Bi, ^226^Ra, ^228^Ac, ^234^Th, ^234m^Pa, ^235^U and ^241^Am. The measurement technique was based on the use of high-purity germanium (HPGe) detectors coupled to the required pulse amplification and shaping electronics and multi-channel analyser (EG&G Ortec, AMETEK Inc.). The γ-ray spectra were stored on a computer and analysed via the software program FitzPeaks Gamma Analysis and Calibration Software (JF Computing Services) for photopeak identification and quantification. The detectors were calibrated for efficiency, energy and peak shape using a certified mixed radionuclide standard, which covers an energy range of approximately 30–2000 keV. The efficiency of γ-rays between 30 and 120 keV was determined on an individual basis. Application of decay corrections for the naturally occurring daughter radionuclides of uranium and thorium assumes that the series daughter radionuclides are all in secular equilibrium and therefore decay with the half-life of the first radionuclide in the series.

### Instrument calibration

All instruments are calibrated using certified standards traceable to national standards. The radioactive controls and internal tracers are also made from certified standards and are supplied by various manufacturers: NPL (UK), Amersham International and National Institute of Standards and Technology (NIST, USA).

### Limit of detection (LoD)

The LoDs were calculated in accordance with International Standard ISO 11929-7. The generic formulae for the detection limit can be simplified by setting a value for the coverage factor (chosen to be 1.645 for 95% probability), and by assuming that the count time is the same as the background count time and that there is negligible relative error in w (u_rel_ (w)). The formula for the limit of detection (*LoD*) in Bq/L or Bq/kg is:

$$LoD = \frac{2.7w}{{t_{s} }} + 4.7w\sqrt {\frac{b}{{t_{s} }}}$$

Where the symbols are defined as follows: b = background count rate (counts/s) (includes continuum when sample present and background when no sample present), t_s_ = sample count time (s), w = 1/(e V f) or 1/(e M f), u_rel_ (w) = total relative standard uncertainties for all the factors making up w.

When calculating the limits of detection in gamma ray spectrometry, it is important to take into account the increased uncertainty from estimating the continuum from a smaller number of channels when peaks are located close together. This is therefore incorporated into the recommended formula above for the peak integration case as follows and in a re-arranged format:

$$LoD = \left( {\frac{{2.71 + 3.29\,\sqrt {\left( {1 + \frac{n}{2m}} \right) \times B} }}{T}} \right) \times w$$

Additional symbols used: *n* = peak width in channels, *m* = number of channels used each side of the peak to determine the continuum.

Where $\left( {\frac{n}{2m}} \right)$ is usually about 1. However if gamma ray peaks are close together and the number of channels available for continuum estimation is reduced then $\left( {\frac{n}{2m}} \right)$ could increase to possibly 4 or more.

A single measurement on each sample was made and a full uncertainty budget calculated as described in the Measurement Good Practice Guide No. 36, British Measurement and Testing Association. The uncertainty is quoted at the 95% confidence level.

#### General comments on LoD

Different LoDs were calculated for different samples of the same analyte; these arise from the factors used in the calculation for the limit of detection in the formula shown above. The values of some factors, such as b, differed from measurement to measurement, resulting in different LoDs for many samples.

The background for most techniques is fairly constant, but this is not the case for analysis by gamma ray spectrometry. Here the individual sample background is the Compton continuum produced by the gamma rays in the spectrum. If, for example, the K-40 level is low in one sample, the Compton continuum will be low and therefore the background will be low. Conversely if the K-40 activity is high, the Compton continuum will be higher and therefore the background will be higher.

### Data presentation and analysis

Measured values for radionuclides in STPs were obtained as measurements of the radioactivity of the sample as received (or wet weight basis, wwb). Values are reported both as activities (mBq/g) and corresponding mass concentrations (g/g) calculated from the specific activities (SA) given in Table 1; the data are presented per gram because STP users commonly use quantities of approximately 1 g or more of snus per application [16]. Mass concentrations allow direct comparison of the data reported here with levels of other chemical toxicants in tobacco. The data are also given on a dry weight basis (dwb), i.e. after the sample weight is adjusted for the water content, as measured by NIR (Additional file 1: Table S1). The wwb values reflect the radionuclide content of the STP as experienced by the user (and measured in this study), whereas the dwb values refer to the radionuclide content of the solid matter of the STP (predominately tobacco) and is reported here to facilitate a comparison both across different types of STP and with published values, which are predominantly reported historically as dwb. Activity data that were originally reported in the literature in units of pCi/g have been converted to mBq/g. Half-lives (τ), SAs and % isotopic compositions were taken from references [17, 18].

Radionuclide levels across categories of different STPs were compared using the General Linear Model ANOVA in Minitab v16. Where reported activity levels were below limits of quantification (LOQ), randomly imputed values between the LOQ and zero (generated using Microsoft Excel 2010) were used for the purpose of these comparisons.

## Results

Although only ^210^Pb, ^210^Po and uranium have been previously reported in STPs, many other radionuclides have been reported to be present in the tobacco plant and tobacco products [8]. The activities of the 28 radionuclides measured in contemporary Swedish snus and US STPs on a wwb are summarised in Tables 2, 3 and 4, with individual product activity values in Additional file 1: Tables S2–S4 and the corresponding mass of these radionuclides presented in Additional file 1: Tables S5–S7. Where available, literature values of the radionuclide concentrations or activities in tobacco products are summarised in Tables 2, 3 and 4.

**Table 2:** Summary of current findings in contemporary STPs and historic values for uranium-235 and radionuclides of the uranium-238 decay series

Radionuclide	Main radioactive emission mode	Proportion of STPs in which radionuclide detected	Range in STPs with detectable levels of radionuclide		Historic radioactivity values in tobacco (mBq/g dwb)^a^	Tobacco type	Country	Date	References
			Radioactivity (mBq/g WWB)	Mass (g/g WWB)					
					8–240 × 10^−9^ g/g	Cigarette	Netherlands	1979	[64]
Total uranium					37–203 × 10^−9^ g/g	Cigarette, chewing, pan	India	1979–1992	[4, 19, 65, 66]
					7.4–19.1 × 10^−6^ g/g	Snuff	India	1985–1987	[4, 19]
^235^U	α	0/70	BLD	BLD	BLQ	Cigarette	India	1979	[65]
					BLD	Snuff, snus	US	2014	[67]
^238^U	α	3/70	1–10	7–80 × 10^−8^	BLD-8.1	Cigarette	Brazil, Egypt	1994–2012	[20, 21]
					BLD	Snuff, snus	US	2014–2016	[67–69]
^234^U	α	5/70	1–9	4–38 × 10^−12^	0.3–1.3	Leaf	Brazil	1994	[20]
^234^Th	β^−^	0/73	BLD	BLD	BLQ	Tobacco	Japan	1987	[70]
					5.7–8.8	Moassel	Egypt	2005	[71]
^234m^Pa	β^−^	0/73	BLD	BLD	None reported				
^230^Th	α	5/70	1–9	1–12 × 10^−12^	None reported				
^226^Ra	α	67/70	1–9	1–24 × 10^−14^	1.8–20	Cigarette, pipe, cigar	Various	1960–2005	[20, 34–36, 71–78]
^214^Pb	β^−^	0/73	BLD	BLD	BLQ	Cigarette	Switzerland	1971	[28]
^214^Bi	α	0/73	BLD	BLD	1.3–2.4	Moassel	Egypt	2005	[71]
					44–59	Tobacco	Iraq	2009	[29]
^210^Pb	β^−^	52/70	< 40	< 2 × 10^−14^	1.9–40	Various	Various	1966–2009	[35, 36, 70, 71, 73, 74, 77, 79–86]
					8.6–11.6	Moist snuff	US	2009	[48]
^210^Po	α	66/70	2–18	1–11 × 10^−17^	0.74–80	Various	Various	1987–1994	[20, 87, 88]
					< 2–120	Snuff, snus	US Sweden	1986–2014	[37, 48, 67, 89]

^a^For total uranium concentrations (g/g) are used, BLD—below limit of detection, BLQ—below limit of quantification

**Table 3:** Summary of current findings in contemporary STPs and historic values for radionuclides of the thorium decay series and for potassium-40 and cosmic ray generated radionuclides

Radionuclide	Main radioactive emission mode	Proportion of measured STPs in which radionuclide detected	Range in STPs with detectable levels of radionuclide		Historic radioactivity values in tobacco (mBq/g dwb)	Tobacco type	Country	Date	References
			Radioactivity (mBq/g WWB)	Mass (g/g WWB)					
^232^Th	α	1/70	1	3 × 10^−7^	0.5–18.4	Various	Various	1973–2012	[20, 21, 90–92]
^228^Ac	β^−^	0/73	BLD	BLD	0.4–6.5	Moassel, cigarette	Egypt, Iraq, Switzerland	1963–2009	[28, 29, 71, 93]
^228^Th	α	47/70	1–9	4–28 × 10^−17^	BLQ-206	Cigarette, leaf	Russia, Brazil	1972–1994	[20, 94]
^212^Pb	β^−^	0/73	BLD	BLD	BLQ-9	Cigarette	Switzerland, Iraq	1971–2009	[28, 29]
^212^Bi	β^−^	0/73	BLD	BLD	9.9–17	Cigarette	Iraq	2009	[29]
^208^Tl	β^−^	0/73	BLD	BLD	BLQ	Cigarette	Switzerland	1971	[28]
^40^K	β^−^	72/73	390–1900	1–7 × 10^−6^	49–2183	Various	Various	1961–2012	[21, 29, 36, 71, 79, 95–100]
^14^C	β^−^	69/73	20–101	1–6 × 10^−13^	None reported				
^3^H	β^−^	2/73	29–65	8–18 × 10^−17^	None reported				

*BLD* below limit of detection, *BLQ* below limit of quantification

**Table 4:** Summary of current findings in contemporary STPs and historic values for anthropogenic radionuclides

Radionuclide	Main radioactive emission mode	Proportion of measured STPs in which radionuclide detected	Range in STPs with detectable levels of radionuclide		Historic radioactivity values in tobacco mBq/g dwb	Tobacco type	Country	Date	References
			Radioactivity (mBq/g WWB)	Mass (g/g WWB)					
^241^Am	α	0/73	BLD	BLD	None reported				
^238^Pu	α	4/73	0.4–1.1	7–17 × 10^−16^	None reported				
^239^Pu	α	7/73	0.3–1.3	1–5 × 10^−13^	0.396 (1960s)–0.005 (1980s)	Cigarette	Finland	1985	[32]
^240^Pu	α			1–4 × 10^−14^					
^137^Cs	β^−^	0/73	BLD	BLD	0.1–40	Various	Various	1983–2012	[21, 35, 98, 99, 101]
^134^Cs	β^−^	0/73	BLD	BLD	None reported				
^131^I	β^−^	0/73	BLD	BLD	None reported				
^60^Co	β^−^	0/73	BLD	BLD	None reported				

*BLD* below limit of detection

### Uranium-235 and radionuclides of the uranium-238 decay series

The activity values of uranium-235 and radionuclides of the uranium-238 decay series are presented in Additional file 1: Table S2, and the corresponding mass concentrations in Additional file 1: Table S5.

Uranium-238 (^238^U, 99.27% of naturally occurring uranium) is a primordial isotope that gives rise to the uranium decay series including uranium-234 (^234^U, 0.0054% of naturally occurring uranium). Uranium-235 (^235^U, 0.72% of naturally occurring uranium) is also a naturally occurring isotope, but is part of the actinium series. In the current work these three radionuclides are discussed together because of the way in which uranium levels have been historically reported, sometimes as total uranium and sometimes as the individual radionuclides.

In the present study ^238^U was detected in only three samples (2 HP, 1 MS) at an activity of 0.8−9.9 mBq/g wwb, ^234^U was detected in 5 products (2 HP, 2 MS, 1 portion snus) at an activity of 0.96–8.8 mBq/g wwb, and ^235^U was not detected in any of the STP samples analyzed (Table 2). In the samples where both ^238^U and ^234^U were present, the two radionuclides had very similar activities; however, owing to the greater specific activity of ^234^U, a substantially greater mass concentration of ^238^U (6.5−80.0 × 10^−8^ g/g wwb) was detected as compared with ^234^U (4–38 × 10^−12^ g/g wwb) (Additional file 1: Table S5).

Referring to Table 2, mass concentrations of *total uranium* have been reported in a variety of cigarettes and chewing tobaccos from the Netherlands and India in the range 8–240 × 10^−9^ g/g, compared with 7.4–19.1 × 10^−6^ g/g in Indian snuff products. The higher levels in Indian snuff was suggested to be due to the inclusion of wood ash/calcium hydroxide [4, 19]. For the individual isotopes ^234^U, ^238^U our results are of the same order of magnitude but slightly higher than those reported for Brazilian and Egyptian tobaccos [20, 21]. Given that the majority of the samples measured in the current study did not have measurable levels of uranium radionuclides, it is of value to estimate the upper limits for their presence in these STPs based on the current analytical capabilities. For the current samples with no measurable uranium radionuclides, the sample dependent upper limits (g/g wwb) were < 3.2−25 × 10^−8^ for ^238^U, < 3–13 × 10^−12^ for ^234^U and < 4−25 × 10^−9^ for ^235^U.

Although there have been two previous reports of thorium-234 (^234^Th) in tobacco, it was not detected in any of the STPs we analysed (Table 2). But because other members of the uranium decay series were identified in all samples, ^234^Th is likely to be present in the STPs albeit at levels below the reporting limits of the analysis (< 20–40 mBq/g wwb, corresponding to < 2.3–4.7 × 10^−17^ g/g wwb).

Protactinium-234 (^234^Pa) has not previously been reported in tobacco products, and none of the STPs we analysed had measurable levels of ^234^Pa (Table 2). The upper limits for activity and mass concentration of ^234^Pa were estimated as < 200–600 mBq/g wwb and < 2.7–8.1 × 10^−18^g/g wwb respectively.

Thorium-230 (^230^Th) has not previously been reported in tobacco. In the present study, five STPs (1 CT, 2 HP, 2 MS) had measurable ^230^Th levels, with an activity of 1–9.2 mBq/g wwb (Table 2), and a mass concentration of 1.0–12.1 × 10^−12^ g/g wwb (Additional file 1: Table S5). Similar to ^234^Th, however, the STPs containing other members of the ^238^U decay series are likely to contain ^230^Th at levels below the reporting limits of the analysis, calculated as < 1–8 × 10^−12^ g/g wwb.

Radium-226 (^226^Ra) was identified in all but three of the samples at an activity of 0.4−8.8 mBq/g wwb (0.5−17.6 mBq/g dwb) (Table 2), corresponding to a mass concentration of 1.1 to 24 × 10^−14^ g/g wwb (1.4−48 × 10^−14^g/g dwb) (Additional file 1: Table S5). ANOVA based comparison of product categories on a wwb showed that ^226^Ra content was similar among most STP categories, except for HP and DS products which had significantly higher levels than MS or CT. On a dry-weight basis, there were generally similar ^226^Ra contents among the STPs analyzed, except that loose and pouched snus had higher levels than CT. Referring to Table 2, several studies have reported ^226^Ra levels in cigarette, pipe and cigar tobaccos from various geographic sources covering a range of 1.8–20 mBq/g dwb, which are comparable to the values found in the present study.

Although lead-214 (^214^Pb) and bismuth-214 (^214^Bi) have previously been reported in tobacco, none of the STPs analyzed showed any ^214^Pb or ^214^Bi (Table 2). The upper limits of activity in the STPs were calculated as < 3–8 and < 3–9 mBq/g wwb respectively, corresponding to a maximum possible content of < 2.5–6.6 × 10^−21^ and < 1.8–5.5 × 10^−21^ g/g wwb respectively.

Among the naturally-occurring radionuclides that become incorporated into tobacco plants, polonium-210 (^210^Po) has received the greatest attention of any radionuclide due to its transfer to smoke in cigarettes [22], and potential for causing lung cancer [23]. In the present study, ^210^Po was detected in 66 of the samples analyzed (Table 2). The measured activities ranged from 1.8−18 mBq/g wwb (3.2−21 mBq/g dwb), corresponding to a mass concentration of 1.1−11 × 10^−17^ g/g wwb (2−13 × 10^−17^ g/g dwb) (Additional file 1: Table S5). Two snus portion products and 2 CT products evaluated in our study were below the detection levels. Comparing the different STP categories the SP and DS products had higher ^210^Po activities (wwb) than the other categories. The ^210^Po content of both loose and pouched snus was lower than the other product categories except for CT. On a dry-weight basis, the DS, MS and SP products had higher ^210^Po activities than the portion, loose snus and CT products. Our results for ^210^Po activities in DS (11.0–17.0 mBq/g wwb) and MS (6.2–9.4 mBq/g wwb) are consistent with previously reported values for these STPs (Table 2).

In the present study, ^210^Pb activities were below the limit of detection of the assay (< 40 mBq/g) for all samples examined. Historic data for ^210^Pb contents of tobacco products are consistent with this (Table 2). A number of authors have reported secular equilibrium between ^210^Pb and ^210^Po due to the length of time between harvesting of tobacco leaves and tobacco product production [24–27]. Consequently, ^210^Pb is likely to be present in the current sample set, at activity levels comparable to the ^210^Po measurements.

### Radionuclides of the thorium-232 decay series

The activity values for thorium-232 decay series radionuclides are presented in Additional file 1: Table S3, and the corresponding mass concentrations in Additional file 1: Table S6.

The primordial isotope thorium-232 (^232^Th) accounts for virtually 100% of the natural abundance of thorium. In the present study, only one US CT product showed a detectable level of ^232^Th (1.1 mBq/g wwb), representing a mass concentration of 0.27 × 10^−6^ g/g wwb (Additional file 1: Table S6). The corresponding dwb values (1.5 mBq/g and 0.37 × 10^−6^ g/g) are in line with levels reported in the literature for total thorium levels (Table 3).

Although the isotope actinium-228 (^228^Ac) is extremely rare (almost all naturally occurring actinium is ^227^Ac), it has been reported in several tobacco samples (Table 3). However, ^228^Ac was not detected in any of the current STPs (Table 3). From the reporting limits, the maximum activity and mass of ^228^Ac that could be present in the STPs were < 8−30 mBq/g wwb (< 16−62 mBq/g dwb) and < 1−3.6 × 10^−19^ g wwb (< 2−7.5 × 10^−19^ g dwb), respectively. The current method is insensitive to the levels of ^228^Ac reported historically of 0.4-6.5 mBq/g (Table 3).

In the current work, 47 of the STPs had detectable levels of ^228^Th with activity ranging from 1.3 to 8.5 mBq/g wwb (2.2−15.8 mBq/g dwb) (Table 3), corresponding to mass concentrations of 4.3–28.0 × 10^−17^ g/g wwb (7.4−52.0 × 10^−17^ g/g dwb) (Additional file 1: Table S6), with many of the measured activities similar in magnitude to the limit of quantification of the analysis. These values are at the lower end of those reported in the literature (Table 3). ANOVA analysis of wwb data showed that DS had higher ^228^Th activity levels than MS products. When expressed as a dry weight basis, there were no significant differences among the product categories.

None of the STPs tested had measurable levels of lead-212 (^212^Pb), bismuth-212 (^212^Bi) or tantalum-208 (^208^Tl) (Table 3). From the reporting limits of the analytical methods, the maximum levels of these nuclides in the STPs were, respectively, < 2−6 mBq/g wwb, < 20−60 mBq/g wwb and < 2−5 mBq/g wwb in terms of activity; and < 3.9−12 × 10^−20^ g/g wwb, < 3.7−11.1 × 10^−20^ g/g wwb and < 1.8−4.6 × 10^−22^ g/g wwb. Although not detected here, trace levels of ^212^Pb and ^208^Tl have been reported in Swiss cigarettes [28], and ^212^Pb and ^212^Bi levels have been quantified [29] in Iraqi cigarettes at 6–9 and 9.9–17 mBq/g, respectively (Table 3).

### Other naturally occurring radionuclides—potassium-40, tritium, carbon-14

For these naturally occuring radionuclides the activity values in the analysed STPs are presented in Additional file 1: Table S3, and the corresponding mass concentrations in Additional file 1: Table S6.

Potassium-40 (^40^K), present at 0.012% of naturally occurring potassium, was identified in all but one of the STPs analyzed (Table 3). The activity levels of 390–1900 mBq/g wwb (419–2145 mBq/g dwb) make it the most radioactive component present in the measured STPs. Furthermore, ^40^K was the radionuclide present at the highest mass concentrations, 1.5–7.2 × 10^−6^ g/g wwb (1.6–8.1 × 10^−6^ g/g dwb); an order of magnitude higher than the next most prevalent radionuclide (Additional file 1: Table S6). These data are within the range of values reported in the literature (49–2183 mBq/g) (Table 3).

**Fig. 1:**
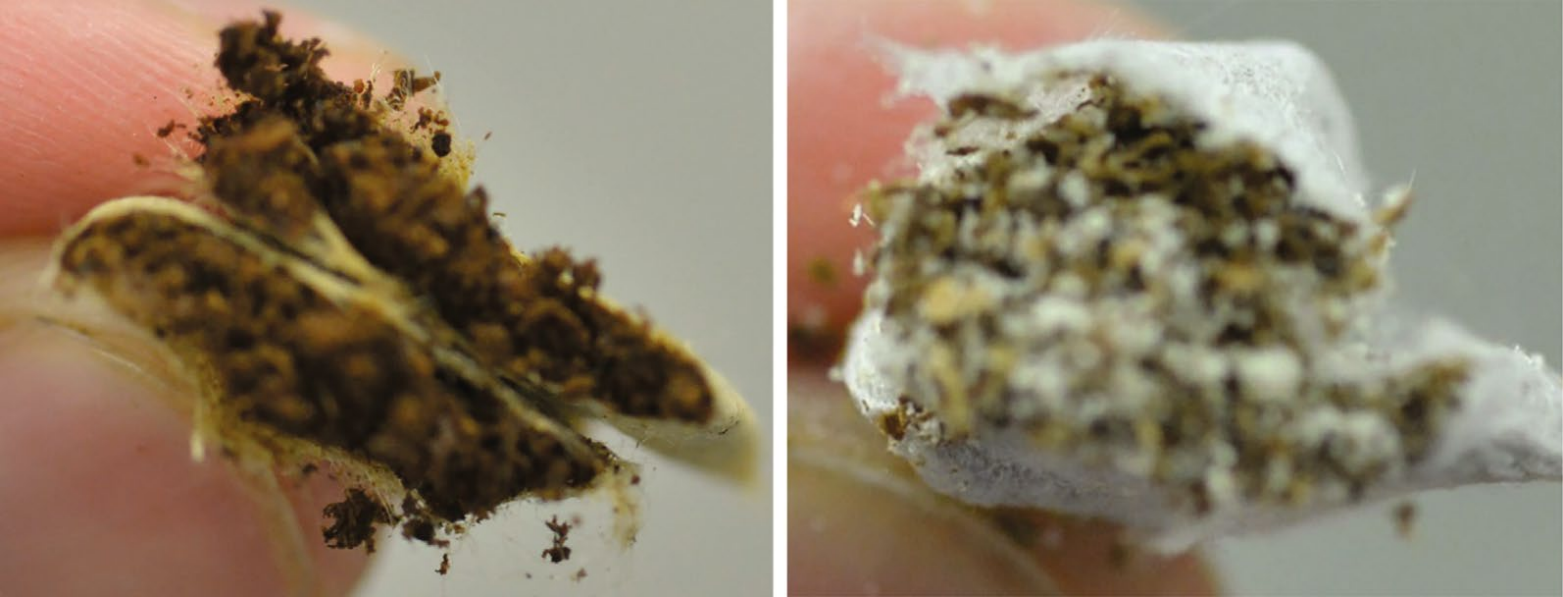
Visual comparison of typical Swedish portion snus (left) and Oomph portion snus (right). Shown are cross-sections of the cut products

Comparing product categories on a wwb showed higher activity levels for DS products, with all other products having similar or lower levels of activity. On a dwb the differences between STP categories diminished, although DS products were still at the higher end of ^40^K content. Activity levels were also lower in CT than in DS and MS products. The sample with no measurable ^40^K content, Oomph (Wise) citrus and menthol (Northerner), is a very dry product with an upper limit of < 0.4 × 10^−6^ g/g ^40^K. Compared to the other snus products the material in the Oomph pouch was lighter in colour and contained a substantial content of a white material (Fig. 1). This was probably due to the cellulose powder and vegetable fibre ingredients reported on the package. The tobacco content comprised 50% of the total product mass. Hence, the lack of detectable ^40^K may well reflect the diluted tobacco content of this STP.

Carbon-14 (^14^C) is largely a product of cosmic ray irradiation of the atmosphere. The ^14^C content of tobaccos has not been reported previously, although an assimilation study [30] has shown that ^14^C is readily taken-up and distributed within the tobacco plant. In the current work, ^14^C was detected in all but one of the STPs, making it one of the more pervasive radionuclides examined in this study. ^14^C activity ranged from 20 to 101 mBq/g wwb (26–135 mBq/g dwb) (Table 3), with a corresponding mass concentration of 1.2 to 5.9 × 10^−13^ g wwb (1.5–8.0 × 10^−13^ g/g dwb) (Additional file 1: Table S6). The product without measurable ^14^C was Romeo y Julieta (Habanos Nordics), a portion snus; for this product, the detection limit was higher than for many of the other STPs; thus, ^14^C may have been present at a level slightly below the limit of detection. Comparison of ^14^C activity levels across different product categories showed no significant differences on either a dwb or wwb.

Although tritium (^3^H) which is also produced by cosmic ray interaction with the atmosphere, has not been reported in tobacco, it was considered a potential contaminant via generation in the atmosphere, and incorporation into the growing tobacco plant as ^3^H-incorporated water. ^3^H was identified in one Swedish snus and one US plug product at up to 65 mBq/g wwb (79 mBq/g dwb) (Table 3) or 1.8 × 10^−16^ g/g wwb (2.2 × 10^−16^ g/g dwb) (Additional file 1: Table S6). The other samples had upper limits for ^3^H activity and content of < 10–43 mBq/g wwb and < 2.8–12 × 10^−17^ g/g wwb, respectively.

Phosphorus-32 (^32^P) is another radionuclide generated by cosmic ray interaction with the atmosphere, and has been categorized by IARC as a Group 1 carcinogen. However, its short half-life (14.3 days) and absence of precursors in the STPs would make its presence in these samples highly unlikely, and was therefore not assayed in the present study.

### Man-made radionuclides

The activity values for man-made radionuclides in the analysed STPs are presented in Additional file 1: Table S4, and the corresponding mass concentrations in Additional file 1: Table S7.

The synthetic radionuclide americium-241 (^241^Am) is generated within nuclear waste. Its presence in tobacco has not been reported previously and it was not found in any of the present STPs analyzed (Table 4). Using the activity reporting limits of < 2–4 mBq/g wwb as upper limits of its potential presence in the STPs, indicated a maximum possible mass concentration of < 1.6–3.2 × 10^−14^ g/g wwb (Additional file 1: Table S7).

The STPs were analyzed for three plutonium radionuclides, ^238^Pu, ^239^Pu and ^240^Pu, which are products of the nuclear reactions of uranium. In the present study, four samples (2 portion snus, 1 CT and 1 MS) had measurable ^238^Pu activities (0.4–1.1 mBq/g wwb; 0.6–2.3 mBq/g dwb) (Table 4) corresponding to 6.9–16.9 × 10^−16^ g/g wwb (8.9–37 × 10^−16^ g/g dwb) (Additional file 1: Table S7). For the samples with no measurable ^238^Pu, the upper limits of activity were estimated as < 0.06–0.7 mBq/g wwb, corresponding to mass concentrations of < 0.95–11 × 10^−16^ g/g wwb. Seven of the STPs (2 portion snus, 3 CT, 2 MS) showed measurable ^239,240^Pu contents with activities of 0.3–1.3 mBq/g wwb (0.6–2.9 mBq/g dwb). For the STPs with no measurable ^239,240^Pu, the upper limits of activity were < 0.06–0.7 mBq/g wwb. The activities of ^239,240^Pu were converted to masses by assuming that ^239^Pu comprises 80% of the total amount of ^239,240^Pu present, which is a balance between the value of approximately 95% ^239^Pu found in weapons-grade plutonium, 84% identified in global atmospheric fall-out and approximately 70–75% ^239^Pu found in reactor-grade plutonium [31]. These values correspond to 7.3–30 × 10^−14^ g/g wwb (14–65 × 10^−14^g/g dwb) of ^239^Pu and 1.8–7.5 10^−14^ g/g wwb (3.5–16.3 × 10^−14^g/g dwb) ^240^Pu.

Among the STPs found to contain plutonium in the present study, the activity levels of ^239,240^Pu were considerably higher than those reported [32] for Finnish cigarette tobaccos in the 1960s (0.396 mBq/g), 1970s (0.012 mBq/g) and 1980s (0.005 mBq/g), which probably reflects different geographic sources of tobacco between the studies.

Both caesium-137 (^137^Cs) and caesium-134 (^134^Cs) are products of nuclear fission reactions, and are contaminants produced in nuclear incidents. In the present study, neither ^137^Cs nor ^134^Cs was detected in any of the STPs examined (Table 4). The upper limits for the possible presence of ^137^Cs and ^134^Cs in the STPs were estimated as < 2–4 mBq/g wwb ^137^Cs and < 2–5 mBq/g wwb ^134^Cs, corresponding to mass concentrations of < 6.2–12.4 × 10^−16^ and < 4.2–10.4 × 10^−17^g/g wwb, respectively. Both ^134^Cs (up to 10 mBq/g) and ^137^Cs (0.1–40 mBq/g) have been reported previously in various tobaccos (Table 4). It has been suggested that geographic source is a determinant of its presence or absence in tobacco [28].

Iodine-131 (^131^I) co-evolves with caesium radionuclides after nuclear reactor incidents. It has not been reported previously in tobacco, probably due to its very short half-life, and it was not detected in any of the current STPs (Table 4).

Cobalt-60 (^60^Co), which is also a product of nuclear fission, was not detected in any of the STPs. From the reporting limits of the method, the maximum possible activity was < 3–10 mBq/g wwb for ^131^I and < 2–6 mBq/g wwb for ^60^Co, corresponding to a mass concentration of < 6.5–21.7 × 10^−19^ g/g wwb and < 5–14 × 10^−17^ g/g wwb, respectively. It has not been reported previously as a natural contaminant in tobacco but has been detected in neutron activated tobacco in laboratory studies [33].

## Discussion

The present study represents the most comprehensive assessment of the radionuclide content of STPs published to date. Seventy-eight contemporary STPs from the USA and Sweden, covering the main product categories and manufacturers, were assessed for the presence of 28 radionuclides, encompassing all of the major sources of environmental radioactivity. Three of the species for which we found quantifiable amounts (^14^C, ^3^H, and ^230^Th) have not previously been reported in tobacco.

### Several radionuclides are present at low levels in STPs

In contrast to the conclusions of recent literature reviews of radionuclides in STPs [1, 6] focusing on ^210^Po, ^235^U and ^238^U, this study has revealed a plurality of radionuclides in contemporary STPs. All STPs were found to contain α- and β-emitting radionuclides [generically categorized by IARC as Group 1 carcinogens when internally deposited—Table 1], and the specific IARC Group 1 carcinogens ^226^Ra, and ^232^Th were identified in a number of STPs. However, none of the radionuclides investigated were detected in all STPs. ^14^C, ^226^Ra, ^210^Po (and by inference, ^210^Pb) and ^40^K were found in almost all (66–69) of the STPs examined, ^228^Th, was identified in over half, and ^3^H, ^238^Pu, ^239,240^Pu, ^238^U, ^234^U, ^232^Th, and ^230^Th, were found in only a few. Other than ^40^K, the mass of radionuclides measured in these STPs were very low in comparison with other toxicants identified in STPs [1, 6], often by many orders of magnitude.

Members of both the ^238^U and ^232^Th decay series were present in the STPs. The most active species from the ^238^U series were ^210^Po (^210^Pb) > (^238^U ~ ^234^U ~ ^230^Th ~ ^226^Ra) respectively in order of activity. For the ^232^Th series, only ^232^Th and ^228^Th were detected, with ^228^Th showing greater activity. Radium-228 (^228^Ra; τ, 5.74 years; SA = 10.1 TBq/g) is a member of the ^232^Th series that was not examined in this study; however, previous reports suggest that it might be present in tobacco at levels similar to or slightly higher than those of the other members of the ^232^Th decay series [34–36].

Radionuclides resulting from cosmic ray irradiation of the atmosphere were also found in the STPs. Particularly notable is the presence of the β-emitter ^14^C, which was found in all but one of the samples examined; ^14^C has not been reported in tobacco before, and it represents a previously unconsidered source of radioactive exposure from tobacco products. ^3^H, also not reported previously in tobacco products, was identified in two STPs. In these two samples, although ^3^H was present at much lower mass concentrations than ^14^C, its radioactivity levels were similar to ^14^C. The substantially lower mass concentrations of ^3^H than ^14^C probably reflect differences in atmospheric production rates and subsequent uptake by the growing tobacco plant. Among the man-made radionuclides examined, some STPs showed measurable quantities of three plutonium radionuclides.

### Many radionuclides are either undetectable or absent from STPs

Although some members of the ^238^U and ^232^Th decay series were present, others (^234^Th, ^234^Pa, ^214^Bi, ^214^Pb, and ^228^Ac, ^212^Pb, ^212^Bi, ^208^Tl respectively), as well as ^235^U, ^131^I and the two caesium radionuclides, showed no activity in any of the STPs examined. Some of these radionuclides have been previously detected in tobacco (^228^Ac, ^214^Bi, ^134^Cs, ^137^Cs, ^214^Pb, ^212^Pb, and ^235^U). When a species was not detected it may be due either to the absence of the species in the analyzed matrix or to insufficient sensitivity of the analytical method for the sample being examined.

There are some indications to the reasons underlying the absence of measured activity from specific radionuclides in some samples. The presence of members of the ^238^U and ^232^Th decay series, particularly the originating radionuclides, in an STP means the presence of other members of the decay series cannot be precluded, albeit at levels below the detection limit of the assay. This is exemplified by the uranium isotopes examined in this study. No STP was found with measurable ^235^U, five samples showed detectable levels of both ^234^U and ^238^U, and two STP samples were found to contain ^234^U but did not have measurable levels of ^238^U. Natural sources of uranium contain these radionuclides at a ratio of 99.27% ^238^U to 0.72% ^235^U to 0.0054% ^234^U; however, ^234^U is the most radioactive uranium isotope, and thus lower concentrations could be detected by the method used in this work. Therefore, ^238^U and ^235^U will also be present, even if not detectable, in the samples containing ^234^U. Moreover, given the very short half-lives of many of the progeny of the ^238^U decay series (such as ^214^Pb and ^214^Bi) it is reasonable to assume that such species may be present, however fleetingly, at some point between production and consumption of an STP.

In contrast, some of the man-made radionuclides with relatively short half-lives (e.g. ^137^Cs, ^134^Cs, ^131^I) were not detected in the STPs, and it is plausible that these species are not present owing to a combination of their decay rates and the age of the tobacco in the STPs post-harvest. The radionuclides ^134^Cs (τ = 2 years) and ^131^I (τ = 8 days) would be expected to have decayed to their progeny in the time scale between recent nuclear reactor incidents (e.g. Chernobyl in 1986) and the date of this study (2008–2010). However, ^137^Cs (τ = 30 years) would have undergone less decay since its emission into the environment following the Chernobyl nuclear accident; therefore, the absence of detectable ^137^Cs probably reflects low levels, if any, absorbed from the environment into the tobaccos used to make these STPs. The analytical method is sufficiently sensitive to detect the levels reported in many of the historical observations, and therefore ^137^Cs may not be present in these STPs. The plutonium radionuclides identified in small numbers of STPs during the present work have half-lives from 87 to 24,000 years. Appreciable quantities of plutonium radionuclides were released into the atmosphere during atmospheric nuclear weapons testing in the mid to last half of the 20th century, and their presence has subsequently been detected in several plant species [32]. However, ^241^Am (τ = 432 years), also a product of man-made nuclear reactions, and a daughter product of ^241^Pu, was not detected in the STPs, but may be present at levels below the sensitivity of the method.

In the present work, upper bounds for the possible presence of undetected radionuclides were calculated from the reporting limits of the activity counting method. For some radionuclides with very short half-lives, the upper reporting limit corresponds to a few atoms of the radionuclide within the STP sample. Notably, no radionuclides were detected with half-lives shorter than 132 days. Conversely, all naturally present radionuclides (other than ^235^U, which, if present in these STPs, would have levels below the sensitivity of the analytical method) with half-lives greater than 132 days were detected in some of the STPs examined in this work. This may either point to an effective cut-off point, based on radionuclide half-life, for the analytical capability of the current approach, or perhaps reflect the age of tobacco at the time of measurement.

### Activity from β-emitters in STPs by far exceed those of α-emitters

**Fig. 2:**
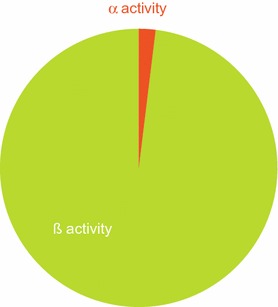
Pie-chart comparing mean α and β activities from STPs

**Fig. 3:**
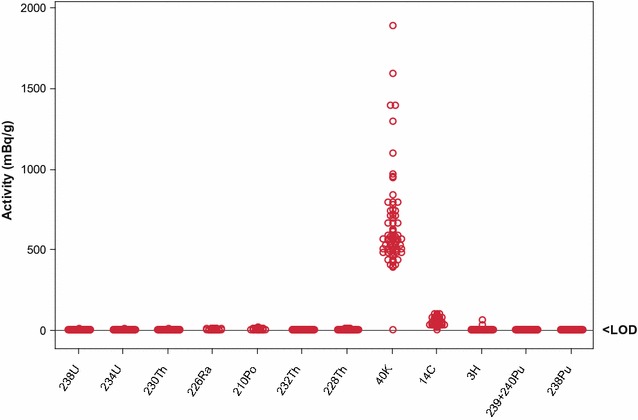
Activities of radioisotopes (mBq/g product wwb) detected in US and Swedish STPs. Activities below LoD are displayed as 0 in the graph

**Fig. 4:**
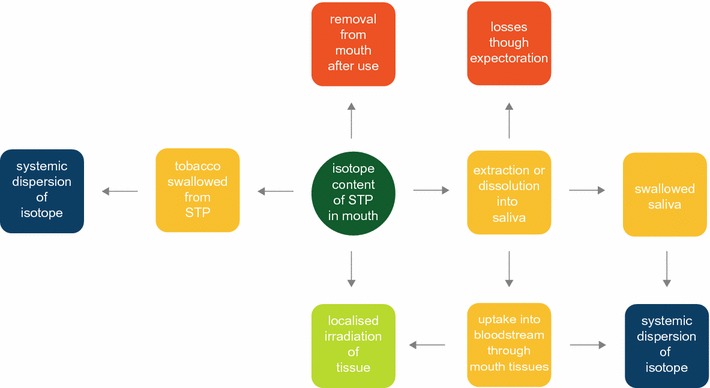
Model for estimating radioactive exposure from isotopes in STPs

The 2008 SCENIHR report [9] stated that “according to Hoffmann et al. [37], the average total activity of alpha emitters in 5 major brands of US snuff was found to be 0.16–1.22 pCi/g” (6–45 mBq/g). Examination of the Hoffmann et al. study [37] reveals the SCENIHR reports statement to be incorrect, and probably an underestimate, in that Hoffmann et al. reported the presence of 0.16–1.22 pCi/g ^210^Po, rather than total α-activity, in 5 US snuff brands. The total α-emissions from the STPs in the current study ranged from 4 to 50 mBq/g wwb and β-emissions ranged from 164 to 1980 mBq/g wwb (plus the unmeasured contribution of ^210^Pb, estimated by comparison to ^210^Po at 1.8–18 mBq/g). Mean values for total α- and total β-emissions are graphically compared in Fig. 2, which clearly shows that total β-emissions are substantially greater than total α-emissions, with β-emissions accounting for 98% on average of the measured activity. Figure 3 shows that in terms of the radioactive emissions from constituents within STPs, the greatest contribution by far was from the β-emitter ^40^K; and, when detectable, the activities of the other β-emitters (^14^C, and ^3^H) were also greater than that of α-emitters. Unlike the potential risk from more volatile radionuclides such as ^210^Po in cigarette tobacco, transfer to smoke is not a factor in assessing exposure to radionuclides in STPs. Among the STPs examined here, the radioactivity of ^210^Po was approximately 1% of that of ^40^K, and therefore ^210^Po is a relatively minor contributor to STP radioactivity. Although as depicted in Fig. 4, and discussed later in more detail, the presence of a given radionuclide in an STP cannot be directly extrapolated to human exposure.

### Radionuclide content varies with STP product type

Some variations in radionuclide content were observed among the different STP categories. Only the HP products had consistently measurable levels of ^238^U, ^234^U and ^230^Th. HP products also had higher levels of ^226^Ra than the other categories on a wwb. The higher levels of these radionuclides likely reflect the presence of non-tobacco (such as calcium carbonate [38]) materials within the HP products. Estimation of the inorganic content (via ashing) of the STPs showed higher inorganic contents in the HP products than in CT, MS, plug, SP, loose snus and all pouched snus other than the low moisture brands. However, the inorganic contents of the DS, dry pouched snus and HP products were comparable. Hence these measurements suggest that the nature of the non-tobacco materials in the HP products may be more important than the quantity. Uranium is known to interchange with calcium in bone samples [39], and the presence of calcium salts in the HP products may act as a source of uranium and daughter radionuclides in STPs.

For the most abundant radionuclide present, ^40^K, the highest levels were found in DS products, and the lowest in an STP whose tobacco content appeared diluted with other materials. No differences were found among product categories for ^14^C or ^228^Th when adjusted for the moisture content of the STPs. The STPs in which ^238^Pu and ^239,240^Pu were detected had similar levels of these man-made radionuclides.

A review of the literature generally indicates that the radionuclides we identified in STPs are similar to levels historically reported in tobacco, except, as noted above, where non-tobacco materials appear to be included in the STP. However, we identified several radionuclides in STPs that have not previously been reported in tobacco.

### Assessing exposure to radionuclides in STPs

#### There is no existing radiological model for evaluating exposure from STPs

Establishing the radionuclide content of STPs is an essential first step in understanding the incremental contribution of radionuclides associated with STP use to the background exposure from radionuclides in our diet, water and air. A key step is to calculate the radiation dose to tissues of STP users, because it allows estimation of the relative risk profiles of different STP product categories, and in principle it facilitates estimation of the risks associated with radionuclides in STPs. Models exist for calculating the radiation dose (exposure energy divided by mass of exposed tissue) resulting from exposure to radionuclides present in our diet, water and air, as well as from occupational exposure (e.g. [40–42]).

However, the type of exposure associated with STP use (shown schematically for use of a generic STP in Fig. 4) is somewhat different to established exposure models. Perhaps the closest established model is that used to calculate the exposure to, and risk from, ingested radionuclides. However, models of ingestion assume rapid mouth transit of the ingested material, and also incorporate the metabolic processes of the body that lead to dispersal of the radionuclide from the gastrointestinal tract to the physiologically preferred accumulation site (e.g. the skeleton for inhaled and ingested uranium radionuclides). STP-use typically involves extended mouth residence (e.g. in the case of Swedish snus an average of 1 h per portion for 12–14 h/day [43]) at habitual locations within the mouth. During this time, the user’s saliva extracts constituents from the STP [16], and the radionuclide-containing saliva may be swallowed or expectorated, but can in principle act as a carrier for radionuclides from STP to mouth tissues for absorption through mucous membranes. During residence in the mouth, radionuclides in the STP may also potentially directly irradiate the tissues adjacent to the STP. Some STPs are dispersed in saliva and not designed to be expectorated; these STPs and their radionuclides will be more readily absorbed or ingested. In those STP categories that are designed for expectoration of the used product, some loose tobacco particles may be swallowed during use. When use of a non-dispersing product is complete, the remaining STP solids (which are heavily loaded with saliva) are removed by the user and discarded.

#### Direct radioactive exposure of oral tissues by STPs is limited

Localized irradiation of the oral tissue of STP users by α- and β-radiation from STPs during use is possible, and Hoffmann et al. [37] suggested that α-radiation emitted by STPs may contribute to an increased risk of snuff dippers for oral cancer due to concentrated irradiation of a relatively small area of the cheek and gum.

However, α-radiation can cause only localized damage owing to its short path length in air and biological matrices (< 0.1 mm) [12], and it is important to note that the dimensions of STP portions are considerably larger than this path length. Therefore only those radionuclides lying very close to the periphery of the STP portion could possibly lead to direct irradiation of oral tissue. For example, we estimate that approximately 1% of the α particles emitted within a snus pouch (i.e. those emitted near the periphery of the portion) would be capable of travelling far enough to exit the snus matrix. In addition, the average thickness of the salivary film, 0.07–0.1 mm [44] will act as an additional barrier to emitted α-radiation, further reducing the likelihood of tissue exposure from α-particles emitted within an STP.

The ability of β-radiation emitted by STP constituents to exit the STP matrix and contact the oral mucosa is highly dependent on the energy of the β-radiation emitted [45]. Low energy β-radiation emitted from ^3^H and ^210^Pb could penetrate only 5–6 μm from the site of emission within the STP, whereas the more energetic β-radiation from ^14^C and ^40^K can potentially penetrate 0.3 and ~ 5 mm respectively [45, 46].

In addition to the barrier properties of the mouth’s salivary film noted above, the outer layer of the oral cavity epithelium, being composed of keratin and subject to continuous sloughing, is a further physical barrier to α- and β-particles. The thickness of the epithelium of the buccal mucosa (the relevant site for STP users) has been measured at around 250 μm in normal healthy subjects [47], and the most superficial keratinized squamous cells are nonvital. It is likely that STP users have somewhat thicker epithelium and a thicker keratin layer, which will physically increase the path length that emitted α- and β-particles must traverse to damage the critical cells in the basal layer. The combination of these factors make it unlikely that biologically-significant damage to oral tissue will result from STP-borne α-emitters and the majority of the β-emitters; however exposure to β-radiation emitted from ^14^C and particularly ^40^K in STPs may be of concern.

These estimations highlight the need for more sophisticated exposure models to assess radiological dose in STP users. These models should consider the committed effective dose arising from exposure to alpha and beta generating radionuclides; internal exposure to alpha radiation is considered more damaging than beta radiation due to the way in which energy is imparted to tissue by these two types of radiation. Several further aspects of direct irradiation need to be considered. First, the main decay mode of many radionuclides that emit α- or β-radiation can be accompanied by gamma radiation emissions. The emitted gamma radiation can introduce an additional radiation dose to the STP user, as gamma radiation can penetrate further and potentially interact with critical biological tissue; this both widens the area of potential radiation exposure but also introduces a relatively low potential for tissue damage due to the comparatively weak interaction of gamma radiation with tissue. Second, there is also potential for bremsstrahlung radiation resulting from interaction of emitted β-radiation with mercury-based dental amalgams in the mouths of some STP users. Some further, potentially important, exposure mechanisms are also important to consider in the development of a model and are described below.

#### Radionuclides can be extracted from STPs by users’ saliva

STP users may also be exposed to radionuclides extracted from the STP by saliva during STP use. Extracted radionuclides may come into closer contact with oral tissues than those remaining within the STP [48], and therefore may more readily expose STP users to radiation. Syed et al. [48] considered ^210^Po extracted in this way to be the main source of irradiation from STPs. However, for most categories of STP (other than dispersable products, for which complete ingestion can be assumed), uncertainties exist over the extent of extraction of individual radionuclides into saliva. There are few data on constituent extraction during STP use, but estimates of the extractability of ^210^Po from US moist snuff in a model system using human saliva was reported as being very low, at 2–10% [48]. There are no data on the extractability of other α-emitters from STPs. It is also difficult to estimate the solubility of these species in tobacco because the exact chemical forms are unknown: recent work has demonstrated that inorganic metalloids in tobacco can be present in multiple chemical states [49] and with differing solubilities [50]. Environmental studies have shown that radium is only moderately soluble in water, but is most soluble under chloride-rich reducing aqueous systems with a high total content of dissolved solids, a condition that might relate to STPs that have a high salt and water content [51]. Environmental thorium has very low aqueous solubility [46]. Aqueous solubilities of uranium, plutonium and neptunium are low but pH dependent [52]. These data suggest limited bioavailability of these α-emitting radionuclides in tobacco, but further studies are required to draw a definitive conclusion.

Regarding the extraction of β-emitters into saliva, a study on the extractability of lead from US moist snuff and Iqmik using artificial saliva showed that lead (and hence ^210^Pb) was not readily extracted (< 8%) from these STPs [53]. Similarly, no measurable level of lead extraction was found during use of snus by US snus consumers [54]. However, ^14^C is incorporated chemically into the tobacco plant in several soluble organic species such as sugars, sugar esters and starches [30], and ^3^H can be present as tritiated water or organic species [41]. Therefore it is likely that these two species would be bioavailable from STPs, although the extent of availability is unclear at present. There are no data on potassium extraction from tobacco; however, a study of the extraction of a range of snus constituents by users showed that ~ 30% of the sodium content was extracted [16]. Because potassium and sodium ions share very similar aqueous solubilities it is plausible to assume that potassium (and hence ^40^K) extractability is also ~ 30% from snus.

Overall, these data suggest that most of the radionuclide content of STPs may remain within the STP during use, but some extraction of radionuclides into saliva, particularly ^40^K, ^3^H and ^14^C, will occur. Once released into saliva, the radiation emitted by saliva-soluble radionuclides will have to overcome the physical shielding effects of saliva, air and non-vital epithelium cells within the oral cavity in order to encounter biologically-important tissue. However, this mechanism does represent a plausible route to the irradiation of STP users’ oral cavities, particularly by ^40^K and ^14^C.

#### Systemic exposure from STP radionuclides

Figure 4 illustrates that systemic dispersion of radionuclides may arise in principle from two routes during STP use: uptake through oral tissues, and swallowing tobacco and tobacco-constituents in saliva.

Radionuclides extracted from STP portions may potentially be absorbed into oral cavity tissues (Fig. 4). If tissue clearance mechanisms are relatively slow compared with STP usage duration, this may lead to a localized build-up of radionuclide in the oral tissue during use, particularly as STP users generally position the tobacco portion at a fixed location within the mouth. However, radiation exposure may be limited in this scenario, as noted above the identified STP radionuclides all have half-life times in excess of 132 days. Standard radiological models do not account for this potential source of exposure, and this is an area requiring further attention.

In contrast, the incremental exposure to radionuclides after swallowing during STP use, is within the scope of the standard radiological dose models for ingested radionuclides from the diet. Systemic dispersion of radionuclides after ingestion is well understood. Potassium (including ^40^K) is almost completely absorbed after ingestion and is quickly distributed to all of the organs and tissues of the body via the bloodstream; it is eliminated from the body with a biological half-life of 30 days. However, the level of potassium in the body is under strict homeostatic control and is not influenced by environmental factors, with an adult male having a body content of 3700 Bq of ^40^K [46]; hence STP use will not increase the body content of ^40^K. Increased exposure to radiation from ^40^K may arise in the GI tract of STP users during transit of swallowed materials; however, comparison to the recommended USA adult daily dietary intake of 4.7 g potassium [55] suggests that GI exposure of STP-sourced ^40^K will be 1–2 orders of magnitude lower than dietary intake. Hence the risk of systemic exposure to ^40^K from STPs will be small. In contrast, STP use can add to the body concentrations of ^3^H, ^14^C, and the progeny of ^238^U and ^232^Th, at levels corresponding to their extractability. Depending upon the effectiveness of fractional absorption from the gut there may also be some GI exposure to radionuclides that undergo extended intestinal transit. The extent of these sources of exposure is unclear, as noted above, but is likely to present a minimal increase in exposure and hence risk in comparison to dietary intake.

#### The risk of radiation exposure from STPs appears low

The greatest potential radiological risk from radionuclides in STPs therefore appears to be from ^40^K, and to a lesser degree ^14^C. Given the localized and extended time of STP use in the mouth, exposure of STP users’ oral tissues to radioactivity may occur either via direct irradiation from within the STP portion or by radionuclides extracted by users’ saliva. With the uncertainties surrounding STP portion size and geometry (and the resulting attenuation of radiation emitted from within STPs), and the differential extent and kinetics of extraction into saliva by users of different STPs, it is challenging to establish an accurate estimate for effective dose to the oral cavity. Clearly, more sophisticated models that account for localized exposure are desirable to quantify radionuclide exposure within the oral cavity, and their development would represent an advance in understanding the potential for oral toxicity of STP use.

Ultimately, epidemiology provides the most informative insights into the risks associated with STP use. Rosenquist et al. [56], Luo et al. [57] and Rodu and Jansson [58] have reviewed the evidence for oral cancer associated with several STP categories. These authors identified no increased risk of oral cancer for snus use by Swedes, and moist snuff and chewing tobacco use by Americans. Assuming that the radionuclide contents of STPs measured in this study are no higher than those present in STPs during the extended time periods corresponding to the epidemiological studies examined in the reviews above, then the levels of radionuclides measured in this study can be regarded as posing no significant hazard to STP users. This conclusion concurs with that expressed in the 2008 SCENIHR report [9] which stated: “the dose of ionising radiation from these sources must be considered as negligible in comparison e.g. with the natural radiation background and other sources of ionising radiations”.

### Regulatory implications of STP radionuclides

The FDA issued a list [5, 59] of harmful or potentially harmful constituents (HPHC) in tobacco products and tobacco smoke, as required by the Federal Food, Drug, and Cosmetic Act (the FD&C Act). The list contains three radionuclides, ^210^Po, ^235^U and ^238^U, and their presence on the list arises [60, 61] from chemical data summarised in IARC Monograph 89 [1], which is in turn based on earlier reviews [62, 63].

However, IARC Monograph 89 (and earlier reviews) contain factual errors relating to these uranium isotopes. Specifically, Table 3 of IARC Monograph 89 (page 58) lists 2.4 pCi/g of ^235^U and 1.91 pCi/g ^238^U in MS, arising from (page 85) a study by Sharma et al. [4] of the uranium content of five Indian snuff products. However, examination of the Sharma et al. study shows that the authors reported no specific data for ^235^U or ^238^U, instead they disclosed specific activity measurements (2.4–6.4 pCi/g) and mass concentrations (7.4–19.1 ppm) for the presence of total uranium [4]. Consequently, the presence of these uranium isotopes on the FDA list is based on flawed data summaries within the IARC monograph.

The findings of this work, which show a more complex picture of STP radiochemistry than previously considered, coupled with errors in IARC Monograph 89, may justify re-examination of the radionuclides currently identified on the FDA HPHC list.

## Conclusion

The present study has revealed a more complete and complex picture of the radionuclide content of STPs than previously reported. 28 radionuclides were examined, covering all four typical sources, of which 13 were detected and quantified in STPs representing 90% market share of the US and Swedish STP markets. A number of radionuclides, such as ^14^C, ^3^H and ^230^Th, are reported in tobacco for the first time.

The most prevalent radionuclides in these STPs were ^40^K, ^14^C, ^210^Po and ^226^Ra, (with ^210^Pb although undetected in these samples, also likely to be widely present due to the secular equilibrium with ^210^Po) Over half the STPs also contained ^228^Th, and 8 radionuclides were identified in a small number of STPs. The activity of β-emitters was much greater than those of α-emitters, and the β-emitter ^40^K was both the most radioactive species and the radionuclide present in the greatest concentration.

In contrast, the three radionuclides identified by the FDA on the HPHC list were either not detected, present in only three of 70 samples, or had activity levels fifty times lower than that of ^40^K. The identities of radionuclides on the HPHC list for smokeless tobacco products may merit reconsideration in the light of these findings.

Critical review of factors potentially leading to exposure of STP users to radioactivity suggests that exposure from alpha emitters may represent minimal risk to STP users, but beta emissions from ^40^K may expose STP user’s oral cavities to levels of radiation during STP use. However, epidemiological evidence suggests that the levels of radionuclides measured in this study appear unlikely to present significant risks to STP users.

## Additional file

**Additional file 1.** Additional tables.

## Abbreviations

CT: chewing tobacco
DS: dry snuff
dwb: dry weight basis
HP: hard pellet
LoD: limit of detection
LoQ: limit of quantification
MS: moist snuff
SA: specific activity
SP: soft pellet
wwb: wet weight (as received) basis
