# Supplementary material for: Comprehensive survey of radionuclides in contemporary smokeless tobacco products
Source: Chem Cent J. 2017 Dec 19;11:131. doi: 10.1186/s13065-017-0359-0 (PMC5735045; doi:10.1186/s13065-017-0359-0)
Supplement: Supplementary file 1 — Additional file 1. Additional tables. [file 13065_2017_359_MOESM1_ESM.docx]

**Table S1: Manufacturer and water content of survey STPs**

| **STP** | **Manufacturer** | **Percentage water content ^a^** | |
| --- | --- | --- | --- |
|  |  | **2008 sample set** | **2010 sample set** |
| **Swedish snus** |  |  |  |
| **Loose snus** |  |  |  |
| Ettan, loose | Swedish Match | 50.7 | 51.2 |
| General, loose | Swedish Match | 49.9 | 50.8 |
| Goteborgs Rape, loose | Swedish Match | 51.9 | 51.7 |
| Granit, loose | Fiedler & Lundgren | 48.7 | 48.6 |
| Grovsnus, loose | Swedish Match | 50.5 | 51.1 |
| Knox, loose | Skruf Snus AB | 51.9 | 51.2 |
| Kronan, loose | Swedish Match | 50.0 | 49.8 |
| LD Original, loose | Japan Tobacco Co. | 50.9 | 50.2 |
| Montecristo, loose | Habanos Nordics | 50.0 | **−** |
| Skruf Stark, loose | Skruf | 52.7 | 49.4 |
| **Portion snus** |  |  |  |
| Catch Licorice, mini portion | Swedish Match | 46.7 | 47.4 |
| Catch White Licorice, portion | Swedish Match | 49.6 | 50.7 |
| CatchDry White Eucalyptus, mini portion | Swedish Match | 29.2 | 19.9 |
| CatchDry White Licorice, mini portion | Swedish Match | 25.1 | **−** |
| Ettan, portion | Swedish Match | 46.4 | 46.9 |
| General, portion | Swedish Match | 48.6 | 46.8 |
| General, mini portion | Swedish Match | 46.5 | 47.3 |
| General White, portion | Swedish Match | 48.7 | 51.1 |
| Goteborgs Rape White, portion | Swedish Match | 49.0 | 50.8 |
| Granit, portion | Fiedler & Lundgren | 49.9 | 48.6 |
| Granit White, portion | Fiedler & Lundgren | 40.7 | 43.6 |
| Grovsnus, portion | Swedish Match | 45.5 | 46.3 |
| Grovsnus White, portion | Swedish Match | 49.9 | 50.5 |
| Gustavus Original, portion | Japan Tobacco Co. | − | 48.7 |
| Knox, portion | Skruf Snus AB | 45.4 | 43.4 |
| Kronan, portion | Swedish Match | 45.3 | 47.0 |
| LD Original, portion | Japan Tobacco Co. | 48.2 | 48.1 |
| Level, portion | Japan Tobacco Co. | 46.8 | **−** |
| Oomph (Wise) Citrus & Menthol, 6 mg | Northerner | 7.4 | 5.0 |
| Romeo y Julieta, portion | Habanos Nordics | 47.7 | 49.2 |
| Skruf Stark, portion | Skruf Snus AB | 48.7 | 43.0 |
| Tre Ankare White, portion | Swedish Match | 51.7 | 50.8 |
| 1847 Original | Philip Morris | 43.8 | **−** |
| 1847 White, portion | Philip Morris | − | 49.8 |
| **American STPs** |  |  |  |
| **Chewing tobacco** |  |  |  |
| Beech Nut | National | 24.1 | 22.5 |
| Chattanooga | Swisher International | 21.1 | 23.6 |
| Durango | National | 23.4 | 23.0 |
| Lancaster | Swisher International | 22.3 | 22.7 |
| Levi Garrett | Conwood | 22.9 | 20.3 |
| Morgans | Conwood | 22.0 | 20.6 |
| Redman Gold | Swedish Match | 25.8 | 23.6 |
| Redman Regular | Swedish Match | 25.3 | 23.2 |
| Southern Pride | Swedish Match | 24.5 | 24.0 |
| Starr | Swisher International | 22.9 | 23.0 |
| Stoker 707 Wintergreen | National | 20.7 | 24.9 |
| Taylors Pride | Conwood | 22.1 | 21.1 |
| Trophy | National | 23.0 | 23.9 |
| **Dry snuff** |  |  |  |
| Bruton | US Smokeless Tobacco Co. | 7.1 | 7.8 |
| Dental Sweet | Conwood | 7.9 | 10.0 |
| Garrett | Conwood | 8.2 | 6.1 |
| Honest | Conwood | 7.3 | 11.1 |
| Square | Swisher | 7.4 | 8.3 |
| **Hard pellet^b^** |  |  |  |
| Ariva Java | Star Scientific Inc.^b^ | 1.8 | 4.5 |
| Stonewall Wintergreen | Star Scientific Inc. | 2.0 | 4.9 |
| **Soft pellet** |  |  |  |
| Oliver Twist Original | House of Oliver Twist | 13.4 | 19.9 |
| **Moist snuff** |  |  |  |
| Copenhagen LC | US Smokeless Tobacco Co. | 52.1 | 50.7 |
| Copenhagen Straight LC | US Smokeless Tobacco Co. | 53.1 | 53.2 |
| Grizzly Natural LC | Conwood | 53.0 | 51.1 |
| Husky Natural FC | US Smokeless Tobacco Co. | 53.9 | 53.6 |
| Husky Straight LC | US Smokeless Tobacco Co. | 54.5 | 54.4 |
| Husky Wintergreen | US Smokeless Tobacco Co. | 52.2 | 52.5 |
| Kayak Straight LC | Swisher | 50.8 | 48.6 |
| Kodiak Straight LC | Conwood | 51.7 | 50.4 |
| Kodiak Wintergreen | Conwood | 49.2 | 49.6 |
| Marlboro Original LC | Philip Morris | 52.1 | − |
| Red Seal Natural FC | US Smokeless Tobacco Co. | 52.4 | 53.6 |
| Red Seal Natural LC | US Smokeless Tobacco Co. | 54.1 | 51.9 |
| Silver Creek | Swisher | 48.8 | 51.6 |
| Skoal Straight | US Smokeless Tobacco Co. | 52.7 | 51.5 |
| Timberwolf Natural FC | Swedish Match | 48.7 | 50.3 |
| Timberwolf Straight LC | Swedish Match | 51.2 | 51.6 |
| **Plug** |  |  |  |
| Cannonball | Conwood | 16.9 | 18.1 |
| **“US snus”** |  |  |  |
| Camel Frost | RJ Reynolds Co. | – | 29.6 |
| Camel Mellow | RJ Reynolds Co. | – | 30.6 |
| Marlboro Mild | Philip Morris | – | 10.4 |
| Marlboro Peppermint | Philip Morris | – | 10.4 |
| Marlboro Rich | Philip Morris | – | 17.7 |
| Marlboro Spearmint | Philip Morris | – | 11.0 |
| ^a^ Water content was determined from NIR values  ^b^ Subsequent to the start of this study, these products have been removed from the market and Star Scientific Inc. has been renamed Rock Creek Pharmaceuticals | | | |

**Table S2. Activity (mBq/g wwb) in contemporary STPs of ^235^U and of radionuclides in the uranium-238 series.**

|  | **Actinium series ^a^** | **Uranium-238 series** | | | | | | | | | |
| --- | --- | --- | --- | --- | --- | --- | --- | --- | --- | --- | --- |
|  | **^235^U** | **^238^U** | **^234^Th** | **^234m^Pa** | **^234^U** | **^230^Th** | **^226^Ra** | **^214^Pb** | **^214^Bi** | **^210^Pb** | **^210^Po** |
| **STP sample set** | 2008^c^/2010^d^ | 2008 | 2010 | 2010 | 2008 | 2008 | 2008^c^/2010^d^ | 2010 | 2010 | 2010 | 2008 |
| **Swedish snus** |  |  |  |  |  |  |  |  |  |  |  |
| **Loose snus** |  |  |  |  |  |  |  |  |  |  |  |
| Ettan | <0.4^c^ | <0.6 | <30 | <300 | <0.6 | <3 | 1.8±0.2^c^ | <4 | <5 | <30 | 3.5±0.8 |
| General | <0.9^c^ | <1 | <20 | <300 | <0.8 | <2 | 8.8±1.4^c^ | <4 | <5 | <30 | 4.0±0.9 |
| Goteborgs Rape | <0.4^c^ | <0.5 | <30 | <400 | <0.6 | <2 | 1.7±0.1^c^ | <5 | <6 | <40 | 3.3±0.8 |
| Granit | <0.6^c^ | <0.6 | <30 | <400 | <0.7 | <1 | 4.6±0.7^c^ | <5 | <6 | <30 | 4.0±1.1 |
| Grovsnus | <0.4^c^ | <0.5 | <30 | <400 | <0.6 | <3 | 1.8±0.2^c^ | <6 | <7 | <40 | 3.5±0.8 |
| Knox | <0.6^c^ | <0.8 | <20 | <200 | <0.9 | <0.8 | 1.6±0.2^c^ | <3 | <3 | <20 | 4.4±0.9 |
| Kronan | <0.3^c^ | <0.7 | <30 | <400 | <0.6 | <4 | 8.6±0.8^c^ | <5 | <7 | <20 | 2.9±0.7 |
| LD Original | <0.8^c^ | <1 | <30 | <200 | <2 | <2 | 2.5±0.2^c^ | <3 | <3 | <20 | 2.7±0.6 |
| Montecristo | <0.4^c^ | <0.6 | –^b^ | – ^b^ | <0.7 | <3 | 1.7±0.2^c^ | – ^b^ | – ^b^ | - | 2.0±0.6 |
| Skruf Stark | <0.4^c^ | <0.6 | <30 | <400 | <0.7 | <0.5 | 1.7±0.2^c^ | <6 | <6 | <30 | 4.6±0.9 |
| **Portion snus** |  |  |  |  |  |  |  |  |  |  |  |
| Catch Licorice, mini portion | <0.7^c^ | <0.9 | <30 | <300 | <0.9 | <3 | 4.0±0.4^c^ | <5 | <6 | <30 | 5.4±1.7 |
| Catch White Licorice, portion | <0.8^c^ | <0.9 | <30 | <400 | <0.8 | <6 | 3.0±0.3^c^ | <5 | <6 | <40 | 5.4±1.6 |
| CatchDry White Eucalyptus, mini portion | <0.6^c^ | <0.8 | <40 | <500 | <0.9 | <2 | 3.4±0.3^c^ | <7 | <8 | <40 | 6.5±1.9 |
| CatchDry White Licorice, mini portion | <0.5^c^ | <0.7 | – | – | <0.7 | <2 | 5.0±0.5^c^ | – | – | <30 | 7.5±2.2 |
| Ettan, portion | <0.4^c^ | <0.6 | <30 | <300 | <0.8 | <4 | 3.4±0.4^c^ | <4 | <5 | <30 | 5.1±1.8 |
| General, portion | <0.8^c^ | <0.8 | <30 | <300 | <0.8 | <3 | 3.2±0.4^c^ | <4 | <5 | <30 | 6.5±2.1 |
| General, mini portion | <0.6^c^ | <0.7 | <30 | <300 | <2 | <3 | 5.2±0.5^c^ | <4 | <5 | <30 | 5.5±1.7 |
| General White, portion | <2^c^ | <2 | <30 | <400 | <2 | <2 | 3.8±0.3^c^ | <6 | <7 | <30 | 3.6±1.4 |
| Goteborgs Rape White, portion | <0.4^c^ | <0.6 | <30 | <300 | <0.8 | <0.8 | 5.6±0.8^c^ | <4 | <5 | <30 | 4.5±1.3 |
| Granit, portion | <0.9^c^ | <2 | <40 | <400 | <2 | <2 | 5.0±0.4^c^ | <5 | <7 | <40 | <4 |
| Granit White, portion | <0.8^c^ | <0.9 | <30 | <400 | <0.9 | <3 | 3.7±0.4^c^ | <5 | <6 | <40 | 4.7±1.3 |
| Grovsnus, portion | <0.4^c^ | <0.5 | <30 | <300 | <0.7 | <2 | 2.9±0.3^c^ | <4 | <5 | <30 | 3.5±1.5 |
| Grovsnus White, portion | <0.3^c^ | <0.4 | <30 | <400 | <0.5 | <2 | 3.5±0.5^c^ | <6 | <7 | <30 | 5.0±1.7 |
| Gustavus Original, portion | <3^d^ | – | <40 | <500 | – | – | <50^d^ | <6 | <7 | <40 | – |
| Knox, portion | <0.3^c^ | <0.4 | <30 | <400 | <0.6 | <2 | 4.0±0.4^c^ | <6 | <6 | <30 | 5.5±1.5 |
| Kronan, portion | <0.3^c^ | <0.5 | <30 | <400 | <0.7 | <2 | 5.3±0.4^c^ | <5 | <6 | <40 | 4.7±1.5 |
| LD Original, portion | <0.3^c^ | <0.4 | <20 | <200 | <0.5 | <4 | 4.6±0.4^c^ | <3 | <4 | <30 | 3.8±1.0 |
| Level, portion | <1^c^ | <1 | – | – | <0.9 | <2 | 5.0±0.4^c^ | – | – | - | 6.1±1.5 |
| Oomph (Wise) Citrus & Menthol, 6 mg | <0.6^c^ | <2 | <30 | <500 | 2.1±0.8 | <3 | 5.1±0.3^c^ | <7 | <8 | <40 | 5.6±1.5 |
| Romeo y Julieta, portion | <0.3^c^ | <0.5 | <40 | <400 | <0.7 | <3 | 3.7±0.4^c^ | <5 | <7 | <40 | <6 |
| Skruf Stark, portion | <0.4^c^ | <0.7 | <30 | <400 | <0.6 | <1 | 3.2±0.4^c^ | <6 | <6 | <30 | 4.5±1.4 |
| Tre Ankare White, portion | <2^c^ | <3 | <30 | <400 | <3 | <1 | 3.9±0.4^c^ | <5 | <6 | <30 | 5.9±1.5 |
| 1847 Original | <0.6^c^ | <0.7 | – | – | <0.7 | <1 | 4.2±0.4 | – | – | - | 1.8±0.8 |
| 1847 White, portion | <3^d^ | – | <30 | <400 | – | – | <40^d^ | <4 | <5 | <30 | – |
| **American STPs** |  |  |  |  |  |  |  |  |  |  |  |
| **Chewing tobacco** |  |  |  |  |  |  |  |  |  |  |  |
| Beech Nut | <0.6^c^ | <2 | <40 | <400 | <1 | <2 | 2.7±0.7^c^ | <5 | <6 | <40 | <8 |
| Chattanooga | <0.8^c^ | <0.9 | <40 | <400 | <2 | <2 | 2.4±0.4^c^ | <5 | <6 | <30 | 4.0±1.2 |
| Durango | <1^c^ | <0.9 | <40 | <400 | <2 | <0.7 | <0.3^c^ | <5 | <6 | <30 | 4.5±1.1 |
| Lancaster | <0.9^c^ | <2 | <40 | <400 | <2 | <2 | 0.4±0.1^c^ | <5 | <6 | <40 | 5.8±1.2 |
| Levi Garrett | <0.9^c^ | <2 | <40 | <400 | <2 | <0.8 | 0.8±0.2^c^ | <5 | <5 | <30 | 6.2±1.3 |
| Morgan’s | <1^c^ | <0.9 | <40 | <400 | <2 | <3 | 2.7±0.4^c^ | <5 | <6 | <30 | 6.2±1.4 |
| Redman Gold | <0.6^c^ | <0.8 | <40 | <400 | <2 | <2 | 6.1±0.5^c^ | <5 | <6 | <30 | 5.7±1.2 |
| Redman Regular | <0.7^c^ | <0.9 | <40 | <400 | <2 | 1.5±0.5 | 1.9±0.3^c^ | <5 | <5 | <40 | 6.0±1.3 |
| Southern Pride | <0.6^c^ | <0.7 | <30 | <400 | <0.9 | <2 | 2.5±0.3^c^ | <4 | <6 | <40 | <8 |
| Starr | <0.5^c^ | <0.6 | <30 | <400 | <0.6 | <2 | 2.1±0.2^c^ | <4 | <6 | <30 | 6.3±1.3 |
| Stoker 707 Wintergreen | <2^c^ | <2 | <30 | <400 | <3 | <0.8 | <0.2^c^ | <4 | <6 | <30 | 7.2±1.4 |
| Taylors Pride | <0.7^c^ | <0.7 | <30 | <400 | <0.8 | <2 | 2.8±0.4^c^ | <5 | <6 | <40 | 10±2 |
| Trophy | <0.7^c^ | <0.9 | <30 | <400 | <0.9 | <0.8 | <0.2^c^ | <5 | <6 | <40 | 5.7±1.3 |
| **Dry snuff** |  |  |  |  |  |  |  |  |  |  |  |
| Bruton | <0.5^c^ | <2 | <40 | <600 | <2 | <3 | 8.6±0.7^c^ | <8 | <9 | <30 | 17±3 |
| Dental Sweet | <2^c^ | <2 | <30 | <500 | <2 | <0.7 | 1.2±0.2^c^ | <7 | <8 | <30 | 11±2 |
| Garrett | <0.8^c^ | <3 | <30 | <500 | <3 | <3 | 5.3±0.5^c^ | <7 | <8 | <30 | 11±3 |
| Honest | <2^c^ | <1 | <40 | <500 | <1 | <0.8 | 5.6±0.6^c^ | <6 | <7 | <40 | 14±2 |
| Square | <1^c^ | <2 | <30 | <500 | <2 | <5 | 6.6±0.5^c^ | <7 | <8 | <30 | 15±4 |
| **Hard pellet** |  |  |  |  |  |  |  |  |  |  |  |
| Ariva Java | <0.9^c^ | 9.9±1.6 | <30 | <400 | 8.8±1.5 | 9.2±1.4 | 7.0±0.6^c^ | <6 | <7 | <30 | 11±3 |
| Stonewall Wintergreen | <2^c^ | 2.9±1.2 | <30 | <300 | 2.6±1.5 | 4.0±1.0 | 5.7±0.5^c^ | <5 | <6 | <30 | 9.8±2.0 |
| **Soft pellet** |  |  |  |  |  |  |  |  |  |  |  |
| Oliver Twist Original | <0.6^c^ | <2 | <30 | <400 | <2 | <1.1 | 5.0±0.4^c^ | <4 | <5 | <30 | 18±5 |
| **Moist snuff** |  |  |  |  |  |  |  |  |  |  |  |
| Copenhagen LC | <0.5^c^ | <0.7 | <30 | <400 | <0.8 | <0.9 | 2.0±0.2^c^ | <5 | <7 | <40 | 6.7±1.3 |
| Copenhagen Straight LC | <0.4^c^ | <0.8 | <30 | <400 | <1 | 1.0±0.3 | 3.0±0.2^c^ | <5 | <6 | <40 | 6.4±1.1 |
| Grizzly Natural LC | <0.4^c^ | <0.8 | <30 | <400 | <0.7 | <0.9 | 2.4±0.2^c^ | <5 | <6 | <40 | 8.8±1.2 |
| Husky Natural FC | <0.4^c^ | <0.6 | <30 | <400 | <0.9 | <0.9 | 2.7±0.3^c^ | <5 | <6 | <40 | 8.0±1.6 |
| Husky Straight LC | <0.4^c^ | <0.9 | <30 | <300 | <0.9 | <0.5 | 2.2 ±0.2^c^ | <4 | <5 | <30 | 6.1±0.9 |
| Husky Wintergreen | <0.7^c^ | <0.8 | <30 | <300 | <1 | <0.4 | 2.3±0.2^c^ | <4 | <5 | <30 | 7.0±1.5 |
| Kayak Straight LC | <0.9^c^ | <1 | <20 | <300 | <2 | <1 | 2.3± 0.2^c^ | <4 | <5 | <30 | 6.2±1.0 |
| Kodiak Straight LC | <0.4^c^ | <1 | <20 | <200 | <0.7 | <0.8 | 2.3± 0.2^c^ | <3 | <4 | <30 | 9.4±2.5 |
| Kodiak Wintergreen | <2^c^ | <2 | <30 | <300 | <2 | <2 | 2.8± 0.2^c^ | <4 | <5 | <30 | 8.1±1.2 |
| Marlboro Original LC | <0.4^c^ | <0.7 | – ^b^ | – ^b^ | <0.6 | <0.8 | 3.0±0.2^c^ | – ^b^ | – ^b^ | -^b^ | 8.5±1.3 |
| Red Seal Natural FC | <0.4^c^ | 0.81±0.33 | <30 | <300 | 0.96±0.43 | 0.8±0.3 | 2.9±0.2^c^ | <4 | <5 | <30 | 7.4±1.1 |
| Red Seal Natural LC | <0.4^c^ | <0.7 | <30 | <300 | <0.8 | <0.5 | 2.4±0.2^c^ | <4 | <5 | <30 | 6.7±1.2 |
| Silver Creek | <0.3^c^ | <0.6 | <30 | <300 | <0.6 | <2 | 2.3± 0.3^c^ | <4 | <5 | <30 | 7.4±1.3 |
| Skoal Straight | <0.5^c^ | <0.7 | <30 | <300 | <0.6 | <0.8 | 3.1±0.4^c^ | <4 | <5 | <30 | 6.9±1.3 |
| Timberwolf Natural FC | <0.7^c^ | <0.5 | <20 | <300 | <0.8 | <0.4 | 2.6±0.2^c^ | <4 | <5 | <30 | 6.7 ±1.1 |
| Timberwolf Straight LC | <0.4^c^ | <0.9 | <30 | <400 | 1.4±0.6 | <0.5 | 2.0±0.2^c^ | <6 | <7 | <30 | 6.8±1.1 |
| **Plug** |  |  |  |  |  |  |  |  |  |  |  |
| Cannonball | <0.5^f^ | <0.8 | <30 | <300 | <0.8 | <2 | 3.4±0.5^c^ | <3 | <4 | <30 | 9.7±1.6 |
| **“US snus”** |  |  |  |  |  |  |  |  |  |  |  |
| Camel Frost | <3^d^ | – ^b^ | <30 | <400 | – ^b^ | – ^b^ | <40^d^ | <6 | <7 | <30 | – ^b^ |
| Camel Mellow | <3^d^ | – ^b^ | <30 | <500 | – ^b^ | – ^b^ | <40^d^ | <6 | <7 | <30 | – ^b^ |
| Marlboro Mild | <3^d^ | – ^b^ | <40 | <600 | – ^b^ | – ^b^ | <50^d^ | <8 | <9 | <30 | – ^b^ |
| Marlboro Peppermint | <4^d^ | – ^b^ | <40 | <400 | – ^b^ | – ^b^ | <50^d^ | <5 | <6 | <40 | – ^b^ |
| Marlboro Rich | <4^d^ | – ^b^ | <40 | <400 | – ^b^ | – ^b^ | <50^d^ | <6 | <6 | <40 | – ^b^ |
| Marlboro Spearmint | <3^d^ | – ^b^ | <40 | <500 | – ^b^ | – ^b^ | <50^d^ | <8 | <9 | <30 | – ^b^ |
| ^a 239^Pu (actinium series) was measured together with ^240^Pu and the data are included in Table 4.  ^b^ - not available and therefore not analysed  ^c^ analysed radiochemically on 2008 samples  ^d^ analysed by gamma radiation spectrometry on 2010 samples | | | | | | | | | | | |

**Table S3: Activity (mBq/g wwb) in contemporary STPs of radionuclides in the thorium series and for potassium-40 and cosmic ray generated radionuclides.**

|  | **Thorium series** | | | | | | **Naturally occurring isotopes** | | |
| --- | --- | --- | --- | --- | --- | --- | --- | --- | --- |
|  | **^232^Th** | **^228^Ac** | **^228^Th** | **^212^Pb** | **^212^Bi** | **^208^Ti** | **^40^K** | **^14^C ^a^** | **^3^H** |
| **STP sample set** | 2008 | 2010 | 2008 | 2010 | 2010 | 2010 | 2010 | 2010 | 2010 |
| **Swedish snus** |  |  |  |  |  |  |  |  |  |
| **Loose snus** |  |  |  |  |  |  |  |  |  |
| Ettan | <0.9 | <20 | <4 | <3 | <30 | <3 | 480±54 | 34±8 | <18 |
| General | <0.7 | <20 | 7.9±1.5 | <3 | <30 | <2 | 440±51 | 32±7 | <16 |
| Goteborgs Rape | <0.5 | <20 | 3.2±0.7 | <4 | <40 | <3 | 410±60 | 46±14 | <18 |
| Granit | <0.5 | <20 | 1.8±0.6 | <4 | <40 | <3 | 480±62 | 58±11 | <16 |
| Grovsnus | <3 | <20 | <4 | <4 | <40 | <3 | 460±72 | 38±9 | <18 |
| Knox | <0.6 | <8 | 2.3±0.6 | <2 | <20 | <2 | 510±44 | 47±11 | <21 |
| Kronan | <2 | <20 | 4.2±1.6 | <4 | <40 | <3 | 390±68 | 29±10 | <11 |
| LD Original | <2 | <10 | <5 | <2 | <20 | <2 | 970±72 | 30±10 | <29 |
| Montecristo | <0.7 | –^b^ | <2 | – ^b^ | – ^b^ | – ^b^ | – ^b^ | – ^b^ | – ^b^ |
| Skruf Stark | <0.6 | <20 | 4.7±0.8 | <4 | <40 | <3 | 490±78 | 23±8 | <24 |
| **Portion snus** |  |  |  |  |  |  |  |  |  |
| Catch Licorice, mini portion | <2 | <20 | <4 | <3 | <30 | <3 | 560±68 | 25±10 | <14 |
| Catch White Licorice, portion | <4 | <20 | <8 | <4 | <40 | <3 | 510±70 | 47±14 | <15 |
| CatchDry White Eucalyptus, mini portion | <2 | <30 | <4 | <5 | <50 | <4 | 800±110 | 64±13 | <22 |
| CatchDry White Licorice, mini portion  **[2008 only]** | <2 | –^b^ | 4.5±1.3 | – ^b^ | – ^b^ | – ^b^ | – ^b^ | – ^b^ | – ^b^ |
| Ettan, portion | <1 | <20 | <5 | <3 | <30 | <3 | 570±60 | 46±11 | <23 |
| General, portion | <0.8 | <20 | 5.0±1.3 | <3 | <30 | <3 | 590±60 | 37±8 | <27 |
| General, mini portion | <0.9 | <20 | <4 | <4 | <30 | <3 | 580±63 | 38±9 | <28 |
| General White, portion | <0.9 | <30 | 3.2±1.0 | <4 | <40 | <3 | 420±69 | 33±8 | <28 |
| Goteborgs Rape White, portion | <0.8 | <20 | 3.4±1.0 | <3 | <30 | <3 | 470±56 | 39±8 | <16 |
| Granit, portion | <0.9 | <30 | <3 | <4 | <40 | <3 | 610±79 | 42±12 | <10 |
| Granit White, portion | <0.7 | <20 | <3 | <4 | <40 | <3 | 570±69 | 74±16 | <22 |
| Grovsnus, portion | <2 | <20 | 4.5±1.3 | <3 | <30 | <3 | 570±62 | 45±11 | <15 |
| Grovsnus White, portion | <2 | <30 | 5.2±1.9 | <4 | <40 | <4 | 530±79 | 31±9 | <14 |
| Gustavus Original, portion | –^b^ | <30 | –^b^ | <5 | <50 | <4 | 1100±120 | 43±14 | <11 |
| Knox, portion | <1 | <20 | 3.3±1.5 | <4 | <40 | <3 | 530±83 | 45±10 | <16 |
| Kronan, portion | <2 | <20 | <5 | <4 | <40 | <3 | 510±70 | 40±10 | <19 |
| LD Original, portion | <2 | <20 | <5 | <3 | <20 | <2 | 960±74 | 32±12 | <15 |
| Level, portion | <0.6 | –^b^ | 4.3±1.1 | –^b^ | –^b^ | –^b^ | –^b^ | –^b^ | –^b^ |
| Oomph (Wise) Citrus & Menthol, 6 mg | <3 | <30 | <4 | <6 | <50 | <4 | <90 | 51±15 | <25 |
| Romeo y Julieta, portion | <0.7 | <30 | <3 | <5 | <40 | <3 | 440±70 | <43 | <19 |
| Skruf Stark, portion | <2 | <20 | 2.9±0.9 | <4 | <40 | <3 | 410±72 | 47±15 | <28 |
| Tre Ankare White, portion | <0.9 | <20 | 3.4±1.2 | <4 | <40 | <3 | 510±68 | 24±10 | <23 |
| 1847 Original | <1 | –^b^ | 3.5±1.2 | –^b^ | –^b^ | –^b^ | –^b^ | –^b^ | –^b^ |
| 1847 White, portion | –^b^ | <20 | –^b^ | <4 | <40 | <3 | 570±70 | 68±15 | 29±10 |
| **American STPs** |  |  |  |  |  |  |  |  |  |
| **Chewing tobacco** |  |  |  |  |  |  |  |  |  |
| Beech Nut | <1 | <20 | 2.7±0.9 | <4 | <40 | <3 | 670±72 | 20 ± 7 | <11 |
| Chattanooga | <0.6 | <20 | <2 | <4 | <40 | <3 | 590±66 | 30 ± 7 | <11 |
| Durango | <0.5 | <20 | 4.4±0.9 | <4 | <40 | <3 | 780±80 | 63±12 | <15 |
| Lancaster | <0.6 | <20 | 1.9±0.6 | <4 | <40 | <3 | 710±73 | 63±14 | <19 |
| Levi Garrett | <0.6 | <20 | 5.0±1.2 | <4 | <40 | <3 | 710±72 | 68±13 | <23 |
| Morgan’s | <2 | <20 | <3 | <4 | <40 | <3 | 530±63 | 70±13 | <16 |
| Redman Gold | <0.8 | <20 | 3.2±1.0 | <4 | <40 | <3 | 620±70 | 62±13 | <17 |
| Redman Regular | 1.1±0.4 | <20 | 3.9±0.9 | <4 | <40 | <3 | 670±73 | 62±16 | <22 |
| Southern Pride | <0.8 | <20 | 1.7±0.8 | <4 | <40 | <3 | 590±70 | 74±13 | <22 |
| Starr | <0.7 | <20 | 5.1±1.0 | <3 | <40 | <3 | 440±60 | 101±14 | <16 |
| Stoker 707 Wintergreen | <0.6 | <20 | 3.2±0.8 | <4 | <40 | <3 | 550±68 | 60±13 | <17 |
| Taylors Pride | <0.8 | <20 | 4.3±1.0 | <4 | <40 | <3 | 700±78 | 74±16 | <20 |
| Trophy | <0.5 | <20 | <3 | <4 | <40 | <3 | 480±64 | 61±13 | <18 |
| **Dry snuff** |  |  |  |  |  |  |  |  |  |
| Bruton | <2 | <30 | 4.5±1.4 | <6 | <60 | <5 | 1600±160 | 94±13 | <26 |
| Dental Sweet | <0.9 | <30 | 8.5±1.6 | <4 | <50 | <4 | 1400±140 | 101±14 | <25 |
| Garrett | <1 | <30 | 6.4±1.5 | <5 | <50 | <4 | 1400±130 | 80±11 | <26 |
| Honest | <0.7 | <30 | 5.1±1.2 | <5 | <50 | <3 | 1900±160 | 75±14 | <25 |
| Square | <3 | <30 | <5 | <4 | <40 | <4 | 1300±140 | 70±14 | <17 |
| **Hard pellet** |  |  |  |  |  |  |  |  |  |
| Ariva Java | <0.7 | <20 | 3.7±0.9 | <4 | <40 | <3 | 400±71 | 73±13 | <22 |
| Stonewall Wintergreen | <1.1 | <20 | 2.2±0.8 | <3 | <30 | <3 | 550±66 | 32±9 | <12 |
| **Soft pellet** |  |  |  |  |  |  |  |  |  |
| Oliver Twist Original | <0.6 | <20 | <2 | <4 | <40 | <3 | 840±82 | 86±16 | <20 |
| **Moist snuff** |  |  |  |  |  |  |  |  |  |
| Copenhagen LC | <0.6 | <20 | 6.0±1.1 | <4 | <40 | <3 | 510±70 | 45±9 | <26 |
| Copenhagen Straight LC | <0.6 | <20 | 4.1±0.7 | <4 | <40 | <3 | 480±64 | 35±10 | <16 |
| Grizzly Natural LC | <0.4 | <20 | 1.8±0.6 | <4 | <40 | <3 | 630±75 | 44±12 | <21 |
| Husky Natural FC | <0.4 | <20 | 2.4±0.5 | <4 | <40 | <3 | 480±66 | 44±12 | <19 |
| Husky Straight LC | <0.5 | <20 | 2.0±0.5 | <3 | <30 | <3 | 540±59 | 43±11 | <20 |
| Husky Wintergreen | <0.3 | <20 | 2.4±0.5 | <3 | <30 | <2 | 570±60 | 37±11 | <18 |
| Kayak Straight LC | <0.4 | <20 | <2 | <3 | <30 | <2 | 530±56 | 40±12 | <17 |
| Kodiak Straight LC | <0.7 | <20 | 2.0±0.5 | <2 | <20 | <2 | 740±60 | 32±10 | <23 |
| Kodiak Wintergreen | <0.9 | <20 | <3 | <3 | <30 | <3 | 740±71 | 58±15 | <19 |
| Marlboro Original LC **[2008 only]** | <0.4 | –^b^ | 2.0±0.4 | –^b^ | –^b^ | –^b^ | –^b^ | –^b^ | –^b^ |
| Red Seal Natural FC | <0.5 | <20 | 3.7±0.6 | <3 | <30 | <3 | 540±57 | 37±10 | <21 |
| Red Seal Natural LC | <0.4 | <20 | 1.4±0.4 | <3 | <30 | <3 | 560±59 | 27±8 | <20 |
| Silver Creek | <1 | <20 | <2 | <3 | <30 | <2 | 580±62 | 33±9 | <43 |
| Skoal Straight | <0.4 | <20 | 2.8±0.7 | <3 | <30 | <2 | 520±58 | 29±9 | <26 |
| Timberwolf Natural FC | <0.6 | <20 | 7.3±1.0 | <3 | <30 | <2 | 490±56 | 30±11 | <30 |
| Timberwolf Straight LC | <0.4 | <30 | 1.3±0.5 | <4 | <40 | <4 | 430±79 | 28±9 | <33 |
| **Plug** |  |  |  |  |  |  |  |  |  |
| Cannonball | <0.7 | <20 | 5.2±1.1 | <3 | <30 | <2 | 670±57 | 33±7 | 65±11 |
| **“US snus”** |  |  |  |  |  |  |  |  |  |
| Camel Frost | –^b^ | <30 | –^b^ | <4 | <40 | <4 | 560±84 | 46±12 | <19 |
| Camel Mellow | –^b^ | <30 | –^b^ | <4 | <40 | <4 | 720±88 | 66±17 | <20 |
| Marlboro Mild | –^b^ | <30 | –^b^ | <5 | <50 | <5 | 800±110 | 81±15 | <21 |
| Marlboro Peppermint | –^b^ | <30 | –^b^ | <4 | <40 | <3 | 800±85 | 80±19 | <27 |
| Marlboro Rich | –^b^ | <20 | –^b^ | <4 | <40 | <3 | 950±100 | 99±17 | <30 |
| Marlboro Spearmint | –^b^ | <30 | –^b^ | <5 | <50 | <5 | 750±110 | 83±18 | <19 |
| ^a^ Analysis is not covered by UKAS accreditation.  ^b^ Sample not available and therefore not analysed | | | | | | | | | |

**Table S4. Activity (mBq/g wwb) in contemporary STPs of anthropogenic radionuclides (2010 data).**

|  | **^241^Am** | **^239+240^Pu** | **^238^Pu** | **^137^Cs** | **^134^Cs** | **^131^I** | **^60^Co** |
| --- | --- | --- | --- | --- | --- | --- | --- |
| **Swedish snus** |  |  |  |  |  |  |  |
| **Loose snus** |  |  |  |  |  |  |  |
| Ettan | <2 | <0.08 | <0.5 | <2 | <3 | <6 | <3 |
| General | <2 | <0.09 | <0.4 | <2 | <3 | <6 | <3 |
| Goteborgs Rape | <3 | <0.4 | <0.5 | <3 | <3 | <7 | <4 |
| Granit | <3 | <0.09 | <0.2 | <3 | <3 | <8 | <4 |
| Grovsnus | <2 | <0.2 | <0.2 | <3 | <4 | <8 | <4 |
| Knox | <2 | <0.3 | <0.4 | <2 | <2 | <4 | <2 |
| Kronan | <2 | <0.3 | <0.2 | <3 | <3 | <5 | <4 |
| LD Original | <2 | <0.2 | <0.09 | <2 | <2 | <3 | <3 |
| Montecristo | –^a^ | –^a^ | –^a^ | –^a^ | –^a^ | –^a^ | –^a^ |
| Skruf Stark | <2 | <0.2 | <0.2 | <3 | <4 | <6 | <4 |
| **Portion snus** |  |  |  |  |  |  |  |
| Catch Licorice, mini portion | <2 | 0.66±0.13 | 0.51±0.13 | <3 | <3 | <4 | <3 |
| Catch White Licorice, portion | <3 | 0.64±0.13 | 0.41±0.11 | <3 | <3 | <7 | <4 |
| CatchDry White Eucalyptus, mini portion | <4 | <0.2 | <0.2 | <4 | <5 | <9 | <5 |
| CatchDry White Licorice, mini portion | –^a^ | –^a^ | –^a^ | –^a^ | –^a^ | –^a^ | –^a^ |
| Ettan, portion | <2 | <0.2 | <0.5 | <2 | <3 | <6 | <3 |
| General, portion | <2 | <0.5 | <0.5 | <2 | <3 | <6 | <3 |
| General, mini portion | <2 | <0.3 | <0.7 | <3 | <3 | <6 | <3 |
| General White, portion | <3 | <0.2 | <0.2 | <3 | <3 | <9 | <4 |
| Goteborgs Rape White, portion | <2 | <0.2 | <0.7 | <2 | <3 | <6 | <3 |
| Granit, portion | <3 | <0.2 | <0.2 | <3 | <4 | <9 | <4 |
| Granit White, portion | <3 | <0.5 | <0.4 | <3 | <3 | <8 | <4 |
| Grovsnus, portion | <2 | <0.1 | <0.5 | <2 | <3 | <6 | <3 |
| Grovsnus White, portion | <3 | <0.3 | <0.4 | <3 | <4 | <8 | <4 |
| Gustavus Original, portion | <3 | <0.3 | <0.4 | <4 | <4 | <10 | <5 |
| Knox, portion | <3 | <0.2 | <0.3 | <3 | <3 | <8 | <4 |
| Kronan, portion | <3 | <0.06 | <0.2 | <3 | <3 | <6 | <3 |
| LD Original, portion | <2 | <0.2 | <0.2 | <2 | <2 | <3 | <3 |
| Level, portion | –^a^ | –^a^ | –^a^ | –^a^ | –^a^ | –^a^ | –^a^ |
| Oomph (Wise) Citrus & Menthol, 6 mg | <3 | <0.2 | <0.2 | <4 | <4 | <7 | <4 |
| Romeo y Julieta, portion | <3 | <0.5 | <0.5 | <4 | <4 | <7 | <4 |
| Skruf Stark, portion | <2 | <0.07 | <0.09 | <3 | <3 | <5 | <4 |
| Tre Ankare White, portion | <3 | <0.2 | <0.2 | <3 | <3 | <6 | <4 |
| 1847 Original | –^a^ | –^a^ | –^a^ | –^a^ | –^a^ | –^a^ | –^a^ |
| 1847 White, portion | <3 | <0.08 | <0.07 | <3 | <3 | <8 | <3 |
| **American STPs** |  |  |  |  |  |  |  |
| **Chewing tobacco** |  |  |  |  |  |  |  |
| Beech Nut | <3 | 0.47±0.12 | 0.44±0.12 | <3 | <3 | <4 | <4 |
| Chattanooga | <3 | <0.5 | <0.2 | <3 | <3 | <4 | <4 |
| Durango | <3 | <0.3 | <0.2 | <3 | <3 | <4 | <4 |
| Lancaster | <3 | <0.2 | <0.2 | <3 | <3 | <4 | <4 |
| Levi Garrett | <3 | <0.4 | <0.2 | <3 | <3 | <4 | <4 |
| Morgan’s | <3 | <0.3 | <0.5 | <3 | <3 | <4 | <3 |
| Redman Gold | <3 | <0.3 | <0.4 | <3 | <3 | <5 | <4 |
| Redman Regular | <3 | 0.50±0.15 | <0.6 | <3 | <3 | <5 | <3 |
| Southern Pride | <3 | <0.4 | <0.3 | <3 | <3 | <4 | <4 |
| Starr | <3 | <0.2 | <0.5 | <3 | <3 | <4 | <4 |
| Stoker 707 Wintergreen | <3 | <0.3 | <0.1 | <3 | <3 | <4 | <4 |
| Taylors Pride | <3 | 0.54±0.14 | <0.5 | <3 | <3 | <4 | <4 |
| Trophy | <3 | <0.3 | <0.5 | <3 | <3 | <4 | <4 |
| **Dry snuff** |  |  |  |  |  |  |  |
| Bruton | <3 | <0.2 | <0.2 | <4 | <5 | <6 | <6 |
| Dental Sweet | <2 | <0.2 | <0.2 | <4 | <4 | <5 | <5 |
| Garrett | <2 | <0.2 | <0.3 | <4 | <4 | <6 | <5 |
| Honest | <3 | <0.7 | <0.5 | <3 | <4 | <6 | <5 |
| Square | <2 | <0.5 | <0.2 | <4 | <4 | <6 | <5 |
| **Hard pellet** |  |  |  |  |  |  |  |
| Ariva Java | <3 | <0.3 | <0.2 | <3 | <3 | <4 | <4 |
| Stonewall Wintergreen | <2 | <0.2 | <0.2 | <3 | <3 | <4 | <3 |
| **Soft pellet** |  |  |  |  |  |  |  |
| Oliver Twist Original | <3 | <0.2 | <0.4 | <3 | <3 | <4 | <4 |
| **Moist snuff** |  |  |  |  |  |  |  |
| Copenhagen LC | <3 | <0.3 | <0.2 | <3 | <3 | <5 | <4 |
| Copenhagen Straight LC | <3 | <0.2 | <0.1 | <3 | <3 | <5 | <4 |
| Grizzly Natural LC | <3 | <0.09 | <0.09 | <3 | <3 | <5 | <4 |
| Husky Natural FC | <3 | <0.09 | <0.08 | <3 | <3 | <5 | <4 |
| Husky Straight LC | <2 | 1.31±0.2 | 1.07±0.18 | <2 | <3 | <3 | <3 |
| Husky Wintergreen | <2 | <0.3 | <0.2 | <2 | <2 | <3 | <3 |
| Kayak Straight LC | <2 | 0.32±0.09 | <0.3 | <2 | <2 | <3 | <3 |
| Kodiak Straight LC | <2 | <0.2 | <0.2 | <2 | <2 | <3 | <2 |
| Kodiak Wintergreen | <2 | <0.08 | <0.2 | <2 | <3 | <4 | <3 |
| Marlboro Original LC | –^a^ | –^a^ | –^a^ | –^a^ | –^a^ | –^a^ | –^a^ |
| Red Seal Natural FC | <2 | <0.2 | <0.4 | <2 | <3 | <4 | <3 |
| Red Seal Natural LC | <2 | <0.2 | <0.2 | <2 | <3 | <4 | <3 |
| Silver Creek | <2 | <0.2 | <0.6 | <2 | <3 | <4 | <3 |
| Skoal Straight | <2 | <0.2 | <0.3 | <2 | <3 | <4 | <3 |
| Timberwolf Natural FC | <2 | <0.2 | <0.06 | <2 | <3 | <4 | <3 |
| Timberwolf Straight LC | <3 | <0.4 | <0.2 | <3 | <3 | <4 | <4 |
| **Plug** |  |  |  |  |  |  |  |
| Cannonball | <2 | <0.3 | <0.2 | <2 | <2 | <3 | <2 |
| **“US snus”** |  |  |  |  |  |  |  |
| Camel Frost | <3 | <0.2 | <0.4 | <3 | <4 | <8 | <4 |
| Camel Mellow | <3 | <0.2 | <0.3 | <3 | <4 | <8 | <4 |
| Marlboro Mild | <3 | <0.3 | <0.2 | <4 | <5 | <10 | <5 |
| Marlboro Peppermint | <3 | <0.2 | <0.4 | <3 | <3 | <7 | <4 |
| Marlboro Rich | <3 | <0.2 | <0.2 | <3 | <3 | <7 | <4 |
| Marlboro Spearmint | <3 | <0.3 | <0.7 | <4 | <5 | <10 | <5 |
| ^a^ sample not available and therefore not analysed | | | | | | | |

**Table S5. Calculated mass concentrations (wwb) in contemporary STPs of ^235^U and of radionuclides in the uranium-238 series.**

|  | **Actinium series** | **Uranium-238 series** | | | | | | | | | |
| --- | --- | --- | --- | --- | --- | --- | --- | --- | --- | --- | --- |
|  | **^235^U**  **(ng g^−1^)** | **^238^U**  **(ng g^−1^)** | **^234^Th**  **(ag g^−1^)** | **^234m^Pa**  **(ag g^−1^)** | **^234^U**  **(pg g^−1^)** | **^230^Th**  **(pg g^−1^)** | **^226^Ra**  **(fg g^−1^)** | **^214^Pb**  **(zg g^−1^)** | **^214^Bi**  **(zg g^−1^)** | **^210^Pb**  **(fg g^−1^)** | **^210^Po**  **(ag g^−1^)** |
| **STP sample set** | 2008^a^/2010^b^ | 2008 | 2010 | 2010 | 2008 | 2008 | 2008^a^/2010^b^ | 2010 | 2010 | 2010 | 2008 |
| **Swedish snus** |  |  |  |  |  |  |  |  |  |  |  |
| **Loose snus** |  |  |  |  |  |  |  |  |  |  |  |
| Ettan | <5 ^a^ | <48 | <34.9 | <4.1 | <3 | <4 | 50±5.6 ^a^ | <3.3 | <3.1 | <11 | 21±5 |
| General | <12 ^a^ | <80 | <23.3 | <4.1 | <4 | <3 | 244±39 ^a^ | <3.3 | <3.1 | <11 | 24±5 |
| Goteborgs Rape | <5 ^a^ | <40 | <34.9 | <5.4 | <3 | <3 | 47±3 ^a^ | <4.1 | <3.7 | <14 | 20±5 |
| Granit | <8 ^a^ | <48 | <34.9 | <5.4 | <3 | <2 | 128±19 ^a^ | <4.1 | <3.7 | <11 | 24±7 |
| Grovsnus | <5 ^a^ | <40 | <34.9 | <5.4 | <3 | <4 | 50±6 ^a^ | <5.0 | <4.3 | <14 | 21±5 |
| Knox | <8 ^a^ | <64 | <23.3 | <2.7 | <4 | <1 | 44±6 ^a^ | <2.5 | <1.8 | <7 | 25±5 |
| Kronan | <4 ^a^ | <56 | <34.9 | <5.4 | <3 | <6 | 239±22 ^a^ | <4.1 | <4.3 | <7 | 17±4 |
| LD Original | <10 ^a^ | <80 | <34.9 | <2.7 | <9 | <3 | 69±6 ^a^ | <2.5 | <1.8 | <7 | 16±4 |
| T. Montecristo | <5 ^a^ | <48 | –^c^ | – ^c^ | <3 | <4 | 47±6 ^a^ | – ^c^ | – ^c^ | - ^c^ | 12±4 |
| Skruf Stark | <5 ^a^ | <48 | <34.9 | <5.4 | <3 | <1 | 47±6 ^a^ | <5.0 | <3.7 | <11 | 28±5 |
| **Portion snus** |  |  |  |  |  |  |  |  |  |  |  |
| Catch Licorice, mini portion | <9 ^a^ | <72 | <34.9 | <4.1 | <4 | <4 | 111±11 ^a^ | <4.1 | <3.7 | <11 | 32±10 |
| Catch White Licorice, portion | <10 ^a^ | <72 | <34.9 | <5.4 | <4 | <8 | 83±8 ^a^ | <4.1 | <3.7 | <14 | 32±10 |
| CatchDry White Eucalyptus, mini p | <8 ^a^ | <64 | <46.5 | <6.8 | <4 | <3 | 94±8 ^a^ | <5.8 | <4.9 | <14 | 39±11 |
| CatchDry White Licorice, mini portion | <7 ^a^ | <56 | − ^c^ | – ^c^ | <3 | <3 | 139±14 ^a^ | – ^c^ | – ^c^ | <11 | 45±13 |
| Ettan, portion | <5 ^a^ | <48 | <34.9 | <4.1 | <4 | <6 | 94±11 ^a^ | <3.3 | <3.1 | <11 | 31±11 |
| General, portion | <10 ^a^ | <64 | <34.9 | <4.1 | <4 | <4 | 89±11 ^a^ | <3.3 | <3.1 | <11 | 39±13 |
| General, mini portion | 8 ^a^ | <56 | <34.9 | <4.1 | <9 | <4 | 144±14 ^a^ | <3.3 | <3.1 | <11 | 33±10 |
| General White, portion | <25 ^a^ | <170 | <34.9 | <5.4 | <9 | <3 | 106±8 ^a^ | <5.0 | <4.3 | <11 | 22±8 |
| Goteborgs Rape White, portion | <5 ^a^ | <48 | <34.9 | <4.1 | <4 | <1 | 156±22 ^a^ | <3.3 | <3.1 | <11 | 27±8 |
| Granit, portion | <12 ^a^ | <170 | <46.5 | <5.4 | <9 | <4 | 139±11 ^a^ | <4.1 | <4.3 | <14 | <24 |
| Granit White, portion | <10 ^a^ | <72 | <34.9 | <5.4 | <4 | <4 | 103±11 ^a^ | <4.1 | <3.7 | <14 | 28±8 |
| Grovsnus, portion | <5 ^a^ | <40 | <34.9 | <4.1 | <3 | <3 | 81±8 ^a^ | <3.3 | <3.1 | <11 | 21±9 |
| Grovsnus White, portion | <4 ^a^ | <32 | <34.9 | <5.4 | <3 | <3 | 97±14 ^a^ | <5.0 | <4.3 | <11 | 30±10 |
| Gustavus Original, portion | (<40^b^) | – | <46.5 | <6.8 | – | – | (<1400^b^) | <5.0 | <4.3 | <14 | – |
| Knox, portion | <4 ^a^ | <32 | <34.9 | <5.4 | <3 | <3 | 111±11 ^a^ | <5.0 | <3.7 | <11 | 33±9 |
| Kronan, portion | <4 ^a^ | <40 | <34.9 | <5.4 | <3 | <3 | 147±11 ^a^ | <4.1 | <3.7 | <14 | 28±9 |
| LD Original, portion | <4 ^a^ | <32 | <23.3 | <2.7 | <3 | <6 | 128±11 ^a^ | <2.5 | <2.5 | <11 | 23±6 |
| Level, portion | <13 ^a^ | <80 | – | – | <4 | <3 | 139±11 ^a^ | – | – | - | 37±9 |
| Oomph (Wise) Citrus & Menthol, 6 mg | <8 ^a^ | <170 | <34.9 | <6.8 | 9.1±3.5 | <4 | 142±8 ^a^ | <5.8 | <4.9 | <14 | 34±9 |
| Romeo y Julieta, portion | <4 ^a^ | <40 | <46.5 | <5.4 | <3 | <4 | 103±11 ^a^ | <4.1 | <4.3 | <14 | <36 |
| Skruf Stark, portion | <5 ^a^ | <56 | <34.9 | <5.4 | <3 | <2 | 89±11 ^a^ | <5.0 | <3.7 | <11 | 27±8 |
| Tre Ankare White, portion | <25 ^a^ | <250 | <34.9 | <5.4 | <13 | <2 | 108±11 ^a^ | <4.1 | <3.7 | <11 | 35±9 |
| 1847 Original | <8 ^a^ | <56 | –^c^ | – ^c^ | <3 | <2 | 117±11 ^a^ | – ^c^ | – ^c^ | - ^c^ | 11±5 |
| 1847 White, portion | (<40^b^) | – ^c^ | <34.9 | <5.4 | – ^c^ | – ^c^ | (<1100^b^) | <3.3 | <3.1 | <11 | – ^c^ |
| **American STPs** |  |  |  |  |  |  |  |  |  |  |  |
| **Chewing tobacco** |  |  |  |  |  |  |  |  |  |  |  |
| Beech Nut | <8 ^a^ | <170 | <46.5 | <5.4 | <5 | <3 | 75±19 ^a^ | <4.1 | <3.7 | <14 | <48 |
| Chattanooga | <10 ^a^ | <88 | <46.5 | <5.4 | <9 | <3 | 67±11 ^a^ | <4.1 | <3.7 | <11 | 24±7 |
| Durango | <13 ^a^ | <72 | <46.5 | <5.4 | <9 | <1 | <9 ^a^ | <4.1 | <3.7 | <11 | 27±7 |
| Lancaster | <12 ^a^ | <170 | <46.5 | <5.4 | <9 | <3 | 11±3 ^a^ | <4.1 | <3.7 | <14 | 35±7 |
| Levi Garrett | <12 ^a^ | <170 | <46.5 | <5.4 | <9 | <1 | 22±4 ^a^ | <4.1 | <3.1 | <11 | 37±8 |
| Morgan’s | <13 ^a^ | <72 | <46.5 | <5.4 | <9 | <4 | 75±11 ^a^ | <4.1 | <3.7 | <11 | 37±8 |
| Redman Gold | <8 ^a^ | <64 | <46.5 | <5.4 | <9 | <3 | 169±14 ^a^ | <4.1 | <3.7 | <11 | 34±7 |
| Redman Regular | <9 ^a^ | <170 | <46.5 | <5.4 | <9 | 2±0.7 | 53±9 ^a^ | <4.1 | <3.1 | <14 | 36±8 |
| Southern Pride | <8 ^a^ | <56 | <34.9 | <5.4 | <4 | <3 | 69±8 ^a^ | <3.3 | <3.7 | <14 | <48 |
| Starr | <7 ^a^ | <48 | <34.9 | <5.4 | <3 | <3 | 58±6 ^a^ | <3.3 | <3.7 | <11 | 38±8 |
| Stoker 707 Wintergreen | <25 ^a^ | <170 | <34.9 | <5.4 | <13 | <1 | <6 ^a^ | <3.3 | <3.7 | <11 | 43±8 |
| Taylors Pride | <9 ^a^ | <56 | <34.9 | <5.4 | <4 | <3 | 78±11 ^a^ | <4.1 | <3.7 | <14 | 60±12 |
| Trophy | <9 ^a^ | <72 | <34.9 | <5.4 | <4 | <1 | <6 ^a^ | <4.1 | <3.7 | <14 | 34±8 |
| **Dry snuff** |  |  |  |  |  |  |  |  |  |  |  |
| Bruton | <7 ^a^ | <170 | <46.5 | <8.1 | <9 | <4 | 240±19 ^a^ | <6.6 | <5.5 | <11 | 102±18 |
| Dental Sweet | <25 ^a^ | <170 | <34.9 | <6.8 | <9 | <1 | 33±6 ^a^ | <5.8 | <4.9 | <11 | 66±12 |
| Garrett | <15 ^a^ | <100 | <34.9 | <6.8 | <9 | <4 | 147±14 ^a^ | <5.8 | <4.9 | <14 | 66±18 |
| Honest | <13 ^a^ | <80 | <34.9 | <6.8 | <7 | <1 | 156±17 ^a^ | <5.0 | <4.3 | <11 | 84±12 |
| Square | <25 ^a^ | <170 | <46.5 | <6.8 | <5 | <7 | 183±14 ^a^ | <5.8 | <4.9 | <11 | 90±24 |
| **Hard pellet** |  |  |  |  |  |  |  |  |  |  |  |
| Ariva Java | <12 ^a^ | 800±130 | <34.9 | <5.4 | 38.2±6.5 | 12.1±1.8 | 194±17 ^a^ | <5.0 | <4.3 | <11 | 66±18 |
| Stonewall Wintergreen | <25 ^a^ | 233±93 | <34.9 | <4.1 | 11.3±6.5 | 5.2±1.3 | 158±14 ^a^ | <4.1 | <3.7 | <11 | 59±12 |
| **Soft pellet** |  |  |  |  |  |  |  |  |  |  |  |
| Oliver Twist Original | <8 ^a^ | <170 | <34.9 | <5.4 | <9 | <2 | 139±11 ^a^ | <3.3 | <3.1 | <11 | 108±30 |
| **Moist snuff** |  |  |  |  |  |  |  |  |  |  |  |
| Copenhagen LC | <7 ^a^ | <56 | <34.9 | <5.4 | <4 | <2 | 56±6 ^a^ | <4.1 | <4.3 | <14 | 40±8 |
| Copenhagen Straight LC | <5 ^a^ | <64 | <34.9 | <5.4 | <5 | 1.3±0.4 | 83±6 ^a^ | <4.1 | <3.7 | <14 | 39±7 |
| Grizzly Natural LC | <5 ^a^ | <64 | <34.9 | <5.4 | <3 | <2 | 67±6 ^a^ | <4.1 | <3.7 | <14 | 53±7 |
| Husky Natural FC | <5 ^a^ | <48 | <34.9 | <5.4 | <4 | <2 | 75±8 ^a^ | <4.1 | <3.7 | <14 | 48±10 |
| Husky Straight LC | <5 ^a^ | <80 | <34.9 | <4.1 | <4 | <1 | 61±6 ^a^ | <3.3 | <3.1 | <11 | 37±5 |
| Husky Wintergreen | <9 ^a^ | <64 | <34.9 | <4.1 | <5 | <1 | 64±6 ^a^ | <3.3 | <3.1 | <11 | 42±9 |
| Kayak Straight LC | <12 ^a^ | <80 | <23.3 | <4.1 | <9 | <2 | 64±6 ^a^ | <3.3 | <3.1 | <11 | 37±6 |
| Kodiak Straight LC | <5 ^a^ | <80 | <23.3 | <2.7 | <3 | <1 | 64±6 ^a^ | <2.5 | <2.5 | <11 | 57±15 |
| Kodiak Wintergreen | <25 ^a^ | <170 | <34.9 | <4.1 | <9 | <3 | 78±6 ^a^ | <3.3 | <3.1 | <11 | 49±7 |
| Marlboro Original LC | <5 ^a^ | <56 | – ^c^ | – ^c^ | <3 | <1 | 83±6 ^a^ | – ^c^ | – ^c^ | - ^c^ | 51±8 |
| Red Seal Natural FC | <5 ^a^ | 65±27 | <34.9 | <4.1 | 4.2±1.9 | 1±0.4 | 81±6 ^a^ | <3.3 | <3.1 | <11 | 45±7 |
| Red Seal Natural LC | <5 ^a^ | <56 | <34.9 | <4.1 | <4 | <1 | 67±6 ^a^ | <3.3 | <3.1 | <11 | 40±7 |
| Silver Creek | <4 ^a^ | <48 | <34.9 | <4.1 | <3 | <3 | 64±8 ^a^ | <3.3 | <3.1 | <11 | 45±8 |
| Skoal Straight | <7 ^a^ | <56 | <34.9 | <4.1 | <3 | <1 | 86±11 ^a^ | <3.3 | <3.1 | <11 | 42±8 |
| Timberwolf Natural FC | <9 ^a^ | <40 | <23.3 | <4.1 | <4 | <1 | 72±6 ^a^ | <3.3 | <3.1 | <11 | 40±7 |
| Timberwolf Straight LC | <5 ^a^ | <72 | <34.9 | <5.4 | 6.1±2.6 | <1 | 56±6 ^a^ | <5.0 | <4.3 | <11 | 41±7 |
| **Plug** |  |  |  |  |  |  |  |  |  |  |  |
| Cannonball | <10 ^a^ | <64 | <34.9 | <4.1 | <4 | <3 | 94±14 ^a^ | <2.5 | <2.5 | <11 | 58±10 |
| **“US snus”** |  |  |  |  |  |  |  |  |  |  |  |
| Camel Frost | – ^c^ | – ^c^ | <34.9 | <5.4 | – ^c^ | – ^c^ | (<1110^b^) | <5.0 | <4.3 | <11 | – ^c^ |
| Camel Mellow | – ^c^ | – ^c^ | <34.9 | <6.8 | – ^c^ | – ^c^ | (<1110^b^) | <5.0 | <4.3 | <11 | – ^c^ |
| Marlboro Mild | – ^c^ | – ^c^ | <46.5 | <8.1 | – ^c^ | – ^c^ | (<1400^b^) | <6.6 | <5.5 | <11 | – ^c^ |
| Marlboro Peppermint | – ^c^ | – ^c^ | <46.5 | <5.4 | – ^c^ | – ^c^ | (<1400^b^) | <4.1 | <3.7 | <14 | – ^c^ |
| Marlboro Rich | – ^c^ | – ^c^ | <46.5 | <5.4 | – ^c^ | – ^c^ | (<1400^b^) | <5.0 | <3.7 | <14 | – ^c^ |
| Marlboro Spearmint | – ^c^ | – ^c^ | <46.5 | <6.8 | – ^c^ | – ^c^ | (<1400^b^) | <6.6 | <5.5 | <11 | – ^c^ |
| ^a^ measurements conducted in 2008  ^b^ measurements conducted in 2010. (2010 measurements were associated with a substantially greater LOD)  ^c^ Samples not available and therefore not analysed. | | | | | | | | | | | |

**Table S6. Calculated mass concentrations (wwb) in contemporary STPs of radionuclides in the thorium series and for potassium-40 and cosmic ray generated radionuclides.**

|  | **Thorium series** | | | | | | **Naturally occurring isotopes** | | |
| --- | --- | --- | --- | --- | --- | --- | --- | --- | --- |
|  | **^232^Th**  **(ng g^−1^)** | **^228^Ac**  **(zg g^−1^)** | **^228^Th**  **(ag g^−1^)** | **^212^Pb**  **(zg g^−1^)** | **^212^Bi**  **(zg g^−1^)** | **^208^Ti**  **(zg g^−1^)** | **^40^K**  **(μg g^−1^)** | **^14^C**  **(fg g^−1^)** | **^3^H**  **(ag g^−1^)** |
| **STP sample set** | 2008 | 2010 | 2008 | 2010 | 2010 | 2010 | 2010 | 2010 | 2010 |
| **Swedish snus** |  |  |  |  |  |  |  |  |  |
| **Loose snus** |  |  |  |  |  |  |  |  |  |
| Ettan | <230 | <242 | <130 | <58 | <55 | <0.3 | 1.81±0.20 | 200±47 | <51 |
| General | <180 | <242 | 260±49 | <58 | <55 | <0.2 | 1.66±0.19 | 188±41 | <45 |
| Goteborgs Rape | <130 | <242 | 110±23 | <78 | <74 | <0.3 | 1.54±0.23 | 271±82 | <51 |
| Granit | <130 | <242 | 59±20 | <78 | <74 | <0.3 | 1.81±0.23 | 341±65 | <45 |
| Grovsnus | <740 | <242 | <130 | <78 | <74 | <0.3 | 1.73±0.27 | 224±53 | <51 |
| Knox | <150 | <96.6 | 76±20 | <39 | <37 | <0.2 | 1.92±0.17 | 276±65 | <59 |
| Kronan | <500 | <242 | 140±53 | <78 | <74 | <0.3 | 1.47±0.26 | 171±59 | <30 |
| LD Original | <500 | <121 | <160 | <39 | <37 | <0.2 | 3.65±0.27 | 176±59 | <81 |
| Montecristo | <180 | –^a^ | <66 | – ^a^ | – ^a^ | – ^a^ | – ^a^ | – ^a^ | – ^a^ |
| Skruf Stark | <150 | <242 | 150±26 | <78 | <74 | <0.3 | 1.85±0.29 | 135±47 | <79 |
| **Portion snus** |  |  |  |  |  |  |  |  |  |
| Catch Licorice, mini portion | <500 | <242 | <130 | <58 | <55 | <0.3 | 2.14±0.26 | 147±59 | <39 |
| Catch White Licorice, portion | <990 | <242 | <260 | <78 | <74 | <0.3 | 1.92±0.26 | 276±82 | <42 |
| CatchDry White Eucalyptus, mini portion | <500 | <362 | <130 | <97 | <92 | <0.4 | 3.01±0.41 | 376±76 | <62 |
| CatchDry White Licorice, mini portion | <500 | – ^a^ | 150±43 | – ^a^ | – ^a^ | – ^a^ | – ^a^ | – ^a^ | – ^a^ |
| Ettan, portion | <250 | <242 | <160 | <58 | <55 | <0.3 | 2.15±0.23 | 271±65 | <65 |
| General, portion | <230 | <242 | <130 | <58 | <55 | <0.3 | 2.22±0.23 | 218±47 | <76 |
| General, mini portion | <200 | <242 | 160±43 | <78 | <55 | <0.3 | 2.19±0.24 | 224±53 | <79 |
| General White, portion | <230 | <362 | 110±33 | <78 | <74 | <0.3 | 1.59±0.26 | 194±47 | <79 |
| Goteborgs Rape White, portion | <200 | <242 | 110±33 | <58 | <55 | <0.3 | 1.77±0.21 | 229±47 | <45 |
| Granit, portion | <230 | <362 | <100 | <78 | <74 | <0.3 | 2.30±0.30 | 247±71 | <28 |
| Granit White, portion | <180 | <242 | <100 | <78 | <74 | <0.3 | 2.15±0.26 | 435±94 | <62 |
| Grovsnus, portion | <500 | <242 | 150±43 | <58 | <55 | <0.3 | 2.15±0.23 | 265±59 | <42 |
| Grovsnus White, portion | <500 | <362 | 170±63 | <78 | <74 | <0.4 | 2.00±0.29 | 182±53 | <39 |
| Gustavus Original, portion | – ^a^ | <362 | – ^a^ | <97 | <92 | <0.4 | 4.14±0.45 | 253±82 | <31 |
| Knox, portion | <250 | <242 | 110±49 | <78 | <74 | <0.3 | 2.00±0.31 | 265±59 | <45 |
| Kronan, portion | <500 | <242 | <160 | <78 | <74 | <0.3 | 1.92±0.26 | 235±59 | <53 |
| LD Original, portion | <500 | <242 | <160 | <58 | <37 | <0.2 | 3.62±0.28 | 188±71 | <42 |
| Level, portion | <150 | – ^a^ | 140±36 | – ^a^ | – ^a^ | – ^a^ | – ^a^ | – ^a^ | – ^a^ |
| Oomph (Wise) Citrus & Menthol, 6 mg | <740 | <362 | <130 | <12 | <92 | <0.4 | <0.34 | 300±88 | <70 |
| Romeo y Julieta, portion | <180 | <362 | <100 | <97 | <74 | <0.3 | 1.66±0.26 | <253 | <53 |
| Skruf Stark, portion | <500 | <242 | 96±30 | <78 | <74 | <0.3 | 1.54±0.27 | 276±88 | <67 |
| Tre Ankare White, portion | <230 | <242 | 110±40 | <78 | <74 | <0.3 | 1.92±0.26 | 141±59 | <65 |
| 1847 Original | <250 | – ^a^ | 120±40 | – ^a^ | – ^a^ | – ^a^ | – ^a^ | – ^a^ | – ^a^ |
| 1847 White, portion | – ^a^ | <242 | – ^a^ | <78 | <74 | <0.3 | 2.15±0.26 | 400±88 | 81.4±28 |
| **American STPs** |  |  |  |  |  |  |  |  |  |
| **Chewing tobacco** |  |  |  |  |  |  |  |  |  |
| Beech Nut | <250 | <242 | 89±30 | <78 | <74 | <0.3 | 2.52±0.27 | 118±41 | <31 |
| Chattanooga | <150 | <242 | <66 | <78 | <74 | <0.3 | 2.22±0.25 | 176±41 | <31 |
| Durango | <130 | <242 | 150±30 | <78 | <74 | <0.3 | 2.94±0.30 | 371±71 | <42 |
| Lancaster | <150 | <242 | 63±20 | <78 | <74 | <0.3 | 2.68±0.28 | 371±82 | <53 |
| Levi Garrett | <150 | <242 | 160±40 | <78 | <74 | <0.3 | 2.68±0.28 | 400±76 | <65 |
| Morgan’s | <500 | <242 | <100 | <78 | <74 | <0.3 | 2.00±0.24 | 412±76 | <45 |
| Redman Gold | <200 | <242 | 110±33 | <78 | <74 | <0.3 | 2.34±0.26 | 365±74 | <48 |
| Redman Regular | 271±99 | <242 | 130±30 | <78 | <74 | <0.3 | 2.52±0.28 | 365±94 | <62 |
| Southern Pride | <200 | <242 | 56±26 | <78 | <74 | <0.3 | 2.22±0.26 | 435±76 | <62 |
| Starr | <180 | <242 | 170±33 | <58 | <74 | <0.3 | 1.66±0.23 | 594±82 | <45 |
| Stoker 707 Wintergreen | <150 | <242 | 110±26 | <78 | <74 | <0.3 | 2.08±0.26 | 353±76 | <48 |
| Taylors Pride | <200 | <242 | 140±33 | <78 | <74 | <0.3 | 2.64±0.29 | 435±94 | <56 |
| Trophy | <130 | <242 | <100 | <78 | <74 | <0.3 | 1.81±0.24 | 359±76 | <51 |
| **Dry snuff** |  |  |  |  |  |  |  |  |  |
| Bruton | <500 | <362 | 150±46 | <12 | <11 | <0.5 | 6.03±0.60 | 553±76 | <73 |
| Dental Sweet | <230 | <362 | 280±53 | <78 | <92 | <0.4 | 5.28±0.53 | 594±82 | <70 |
| Garrett | <250 | <362 | 210±49 | <97 | <92 | <0.4 | 5.30±0.49 | 471±65 | <73 |
| Honest | <180 | <362 | 170±40 | <78 | <74 | <0.4 | 7.16±0.60 | 441±82 | <70 |
| Square | <740 | <362 | <160 | <97 | <92 | <0.3 | 4.89±0.53 | 412±82 | <48 |
| **Hard pellet** |  |  |  |  |  |  |  |  |  |
| Ariva Java | <180 | <242 | 120±30 | <78 | <74 | <0.3 | 1.51±0.27 | 429±76 | <62 |
| Stonewall Wintergreen | <280 | <242 | 73±26 | <58 | <55 | <0.3 | 2.07±0.25 | 188±53 | <34 |
| **Soft pellet** |  |  |  |  |  |  |  |  |  |
| Oliver Twist Original | <150 | <242 | <66 | <78 | <74 | <0.3 | 3.16±0.31 | 506±94 | <56 |
| **Moist snuff** |  |  |  | <78 |  |  |  |  |  |
| Copenhagen LC | <150 | <242 | 200±36 | <78 | <74 | <0.3 | 1.92±0.26 | 265±53 | <73 |
| Copenhagen Straight LC | <150 | <242 | 140±23 | <78 | <74 | <0.3 | 1.81±0.24 | 206±59 | <45 |
| Grizzly Natural LC | <100 | <242 | 59±20 | <78 | <74 | <0.3 | 2.37±0.28 | 259±71 | <59 |
| Husky Natural FC | <100 | <242 | 79±16 | <78 | <74 | <0.3 | 1.81±0.25 | 259±71 | <53 |
| Husky Straight LC | <130 | <242 | 66±16 | <58 | <55 | <0.3 | 2.03±0.22 | 253±65 | <56 |
| Husky Wintergreen | <74 | <242 | 79±16 | <58 | <55 | <0.2 | 2.15±0.23 | 218±65 | <51 |
| Kayak Straight LC | <100 | <242 | <66 | <58 | <55 | <0.2 | 2.00±0.21 | 235±71 | <48 |
| Kodiak Straight LC | <180 | <242 | 66±16 | <39 | <37 | <0.2 | 2.79±0.23 | 188±59 | <65 |
| Kodiak Wintergreen | <230 | <242 | <100 | <58 | <55 | <0.3 | 2.70±0.27 | 341±88 | <53 |
| Marlboro Original LC | <100 | – ^a^ | 66±13 | – ^a^ | – ^a^ | – ^a^ | – ^a^ | – ^a^ | – ^a^ |
| Red Seal Natural FC | <130 | <242 | 120±20 | <58 | <55 | <0.3 | 2.03±0.21 | 218±59 | <59 |
| Red Seal Natural LC | <100 | <242 | 46±13 | <58 | <55 | <0.3 | 2.11±0.22 | 159±47 | <56 |
| Silver Creek | <250 | <242 | <66 | <58 | <55 | <0.2 | 2.19±0.23 | 194±53 | <121 |
| Skoal Straight | <100 | <242 | 92±23 | <58 | <55 | <0.2 | 1.96±0.22 | 1.71±53 | <73 |
| Timberwolf Natural FC | <150 | <242 | 240±33 | <58 | <55 | <0.2 | 1.85±0.21 | 1.76±65 | <84 |
| Timberwolf Straight LC | <100 | <362 | 43±16 | <78 | <74 | <0.4 | 1.62±0.30 | 1.65±53 | <93 |
| **Plug** |  |  |  |  |  |  |  |  |  |
| Cannonball | <180 | <242 | 170±36 | <58 | <55 | <0.2 | 2.52±0.21 | 1.94±41 | 182 |
| **“US snus”** |  |  |  |  |  |  |  |  |  |
| Camel Frost | – ^a^ | <362 | – ^a^ | <78 | <74 | <0.4 | 2.11±0.32 | 271±71 | <53 |
| Camel Mellow | – ^a^ | <362 | – ^a^ | <78 | <74 | <0.4 | 2.71±0.33 | 388±100 | <56 |
| Marlboro Mild | – ^a^ | <362 | – ^a^ | <97 | <92 | <0.5 | 3.01±0.41 | 476±88 | <59 |
| Marlboro Peppermint | – ^a^ | <362 | – ^a^ | <78 | <74 | <0.3 | 3.01±0.32 | 471±112 | <76 |
| Marlboro Rich | – ^a^ | <242 | – ^a^ | <78 | <74 | <0.3 | 3.58±0.38 | 582±100 | <84 |
| Marlboro Spearmint | – ^a^ | <362 | – ^a^ | <97 | <92 | <0.5 | 2.83±0.41 | 488±106 | <53 |
| ^a^ samples not available and therefore not analysed | | | | | | | | | |

**Table S7. Calculated mass concentration (wwb) in contemporary STPs of anthropogenic radionuclides (2010 data).**

|  | **^241^Am**  **(fg g^−1^)** | **^238^Pu**  **(fg g^−1^)** | **^239^Pu**  **(fg g^−1^)** | **^240^Pu**  **(fg g^−1^)** | **^137^Cs**  **(fg g^−1^)** | **^134^Cs**  **(fg g^−1^)** | **^131^I**  **(ag g^−1^)** | **^60^Co**  **(fg g^−1^)** |
| --- | --- | --- | --- | --- | --- | --- | --- | --- |
| **Swedish snus** |  |  |  |  |  |  |  |  |
| **Loose snus** |  |  |  |  |  |  |  |  |
| Ettan | <15.8 | <0.1 | <4.5 | <1.1 | <0.6 | <0.06 | <1.3 | <0.07 |
| General | <15.8 | <0.6 | <5.1 | <1.3 | <0.6 | <0.06 | <1.3 | <0.07 |
| Goteborgs Rape | <23.6 | <0.8 | <22.7 | <5.7 | <0.9 | <0.06 | <1.5 | <0.10 |
| Granit | <23.6 | <0.3 | <5.1 | <1.3 | <0.9 | <0.06 | <1.7 | <0.10 |
| Grovsnus | <15.8 | <0.3 | <11.4 | <2.8 | <0.9 | <0.08 | <1.7 | <0.10 |
| Knox | <15.8 | <0.6 | <17.1 | <4.3 | <0.6 | <0.04 | <0.9 | <0.05 |
| Kronan | <15.8 | <0.3 | <17.1 | <4.3 | <0.9 | <0.06 | <1.1 | <0.10 |
| LD Original | <15.8 | <0.1 | <11.4 | <2.8 | <0.6 | <0.04 | <0.5 | <0.07 |
| Montecristo | –^a^ | –^a^ | –^a^ | –^a^ | –^a^ | –^a^ | –^a^ | –^a^ |
| Skruf Stark | <15.8 | <0.3 | <11.4 | <2.8 | <0.9 | <0.08 | <1.3 | <0.10 |
| **Portion snus** |  |  |  |  |  |  |  |  |
| Catch Licorice, mini | <15.8 | 0.8 | 37.5 | 9.4 | <0.9 | <0.06 | <0.9 | <0.07 |
| Catch White Licorice | <23.6 | 1.0 | 36.4 | 9.1 | <0.9 | <0.06 | <1.5 | <0.10 |
| CatchDry White Eucalyptus, mini | <31.5 | <0.3 | <11.4 | <2.8 | <1.2 | <0.10 | <2.0 | <0.12 |
| CatchDry White Licorice, mini | –^a^ | –^a^ | –^a^ | –^a^ | –^a^ | –^a^ | –^a^ | –^a^ |
| Ettan | <15.8 | <0.8 | <11.4 | <2.8 | <0.6 | <0.06 | <1.3 | <0.07 |
| General | <15.8 | <0.8 | <28.4 | <7.1 | <0.6 | <0.06 | <1.3 | <0.07 |
| General, mini | <15.8 | <1.1 | <17.1 | <4.3 | <0.9 | <0.06 | <1.3 | <0.07 |
| General White | <23.6 | <0.3 | <11.4 | <2.8 | <0.9 | <0.06 | <2.0 | <0.10 |
| Goteborgs Rape White | <15.8 | <1.1 | <11.4 | <2.8 | <0.6 | <0.06 | <1.3 | <0.07 |
| Granit | <23.6 | <0.3 | <11.4 | <2.8 | <0.9 | <0.08 | <2.0 | <0.10 |
| Granit White | <23.6 | <0.6 | <28.4 | <7.1 | <0.9 | <0.06 | <1.7 | <0.10 |
| Grovsnus | <15.8 | <0.8 | <5.7 | <1.4 | <0.6 | <0.06 | <1.3 | <0.07 |
| Grovsnus White | <23.6 | <0.6 | <17.1 | <4.3 | <0.9 | <0.08 | <1.7 | <0.10 |
| Gustavus Original | <23.6 | <0.6 | <17.1 | <4.3 | <1.2 | <0.08 | <2.2 | <0.12 |
| Knox | <23.6 | <0.5 | <11.4 | <2.8 | <0.9 | <0.06 | <1.7 | <0.10 |
| Kronan | <23.6 | <0.3 | <3.4 | <0.9 | <0.9 | <0.06 | <1.3 | <0.07 |
| LD Original | <15.8 | <0.3 | <11.4 | <2.8 | <0.6 | <0.04 | <0.7 | <0.07 |
| Level | –^a^ | –^a^ | –^a^ | –^a^ | –^a^ | –^a^ | –^a^ | –^a^ |
| Oomph (Wise) Citrus & Menthol, 6 mg | <23.6 | <0.3 | <11.4 | <2.8 | <1.2 | <0.08 | <1.5 | <0.10 |
| Romeo y Julieta | <23.6 | <0.8 | <28.4 | <7.1 | <1.2 | <0.08 | <1.5 | <0.10 |
| Skruf Stark | <15.8 | <0.1 | <4.0 | <1.0 | <0.9 | <0.06 | <1.1 | <0.10 |
| Tre Ankare White | <23.6 | <0.3 | <11.4 | <2.8 | <0.9 | <0.06 | <1.3 | <0.10 |
| 1847 Original | –^a^ | –^a^ | –^a^ | –^a^ | –^a^ | –^a^ | –^a^ | –^a^ |
| 1847 White | <23.6 | <0.1 | <4.5 | <1.1 | <0.9 | <0.06 | <1.7 | <0.07 |
| **American STPs** |  |  |  |  |  |  |  |  |
| **Chewing tobacco** |  |  |  |  |  |  |  |  |
| Beech Nut | <23.6 | <0.7 | 26.7 | 6.7 | <0.9 | <0.06 | <0.9 | <0.10 |
| Chattanooga | <23.6 | <0.3 | <28.4 | <7.1 | <0.9 | <0.06 | <0.9 | <0.10 |
| Durango | <23.6 | <0.3 | <17.1 | <4.3 | <0.9 | <0.06 | <0.9 | <0.10 |
| Lancaster | <23.6 | <0.3 | <11.4 | <2.8 | <0.9 | <0.06 | <0.9 | <0.10 |
| Levi Garrett | <23.6 | <0.3 | <22.7 | <5.7 | <0.9 | <0.06 | <0.9 | <0.10 |
| Morgan’s | <23.6 | <0.5 | <17.1 | <4.3 | <0.9 | <0.06 | <0.9 | <0.07 |
| Redman Gold | <23.6 | <0.6 | <17.1 | <4.3 | <0.9 | <0.06 | <1.1 | <0.10 |
| Redman Regular | <23.6 | <0.9 | 28.4 | 7.1 | <0.9 | <0.06 | <1.1 | <0.07 |
| Southern Pride | <23.6 | <0.5 | <22.7 | <5.7 | <0.9 | <0.06 | <0.9 | <0.10 |
| Starr | <23.6 | <0.8 | <11.4 | <2.8 | <0.9 | <0.06 | <0.9 | <0.10 |
| Stoker 707 Wintergreen | <23.6 | <0.5 | <17.1 | <4.3 | <0.9 | <0.06 | <0.9 | <0.10 |
| Taylors Pride | <23.6 | <0.8 | 30.7 | 7.7 | <0.9 | <0.06 | <0.9 | <0.10 |
| Trophy | <23.6 | <0.8 | <17.1 | <4.3 | <0.9 | <0.06 | <0.9 | <0.10 |
| **Dry snuff** |  |  |  |  |  |  |  |  |
| Bruton | <23.6 | <0.3 | <11.4 | <2.8 | <1.2 | <0.104 | <1.3 | <0.14 |
| Dental Sweet | <15.8 | <0.3 | <11.4 | <2.8 | <1.2 | <0.08 | <1.1 | <0.12 |
| Garrett | <15.8 | <0.5 | <11.4 | <2.8 | <1.2 | <0.08 | <1.3 | <0.12 |
| Honest | <15.8 | <0.8 | <39.8 | <9.9 | <1.2 | <0.08 | <1.3 | <0.12 |
| Square | <23.6 | <0.3 | <28.4 | <7.1 | <0.9 | <0.08 | <1.3 | <0.12 |
| **Hard pellet** |  |  |  |  |  |  |  |  |
| Ariva Java | <23.6 | <0.3 | <17.1 | <4.3 | <0.9 | <0.06 | <0.9 | <0.10 |
| Stonewall Wintergreen | <15.8 | <0.3 | <11.4 | <2.8 | <0.9 | <0.06 | <0.9 | <0.07 |
| **Soft pellet** |  |  |  |  |  |  |  |  |
| Oliver Twist Original | <23.6 | <0.6 | <11.4 | <2.8 | <0.9 | <0.06 | <0.9 | <0.10 |
| **Moist snuff** | . |  |  |  |  |  |  |  |
| Copenhagen LC | <23.6 | <0.3 | <17.1 | <4.3 | <0.9 | <0.06 | <1.1 | <0.10 |
| Copenhagen Straight LC | <23.6 | <0.2 | <11.4 | <2.8 | <0.9 | <0.06 | <1.1 | <0.10 |
| Grizzly Natural LC | <23.6 | <0.1 | <5.1 | <1.3 | <0.9 | <0.06 | <1.1 | <0.10 |
| Husky Natural FC | <23.6 | <0.1 | <5.1 | <1.3 | <0.9 | <0.06 | <1.1 | <0.10 |
| Husky Straight LC | <15.8 | 1.69 | 74.5 | 18.6 | <0.6 | <0.06 | <0.7 | <0.07 |
| Husky Wintergreen | <15.8 | <0.3 | <17.1 | <4.3 | <0.6 | <0.04 | <0.7 | <0.07 |
| Kayak Straight LC | <15.8 | <0.5 | 18.2 | 4.5 | <0.6 | <0.04 | <0.7 | <0.07 |
| Kodiak Straight LC | <15.8 | <0.3 | <11.4 | <2.8 | <0.6 | <0.04 | <0.7 | <0.48 |
| Kodiak Wintergreen | <15.8 | <0.3 | <4.5 | <1.1 | <0.6 | <0.06 | <0.9 | <0.07 |
| Marlboro Original LC | –^a^ | –^a^ | –^a^ | –^a^ | –^a^ | –^a^ | –^a^ | –^a^ |
| Red Seal Natural FC | <15.8 | <0.6 | <11.4 | <2.8 | <0.6 | <0.06 | <0.9 | <0.07 |
| Red Seal Natural LC | <15.8 | <0.3 | <11.4 | <2.8 | <0.6 | <0.06 | <0.9 | <0.07 |
| Silver Creek | <15.8 | <0.9 | <11.4 | <2.8 | <0.6 | <0.06 | <0.9 | <0.07 |
| Skoal Straight | <15.8 | <0.5 | <11.4 | <2.8 | <0.6 | <0.06 | <0.9 | <0.07 |
| Timberwolf Natural FC | <15.8 | <0.9 | <11.4 | <2.8 | <0.6 | <0.06 | <0.9 | <0.07 |
| Timberwolf Straight LC | <23.6 | <0.3 | <22.7 | <5.7 | <0.9 | <0.06 | <0.9 | <0.10 |
| **Plug** |  |  |  |  |  |  |  |  |
| Cannonball | <15.8 | <0.3 | <17.1 | <4.3 | <0.6 | <0.04 | <0.7 | <0.48 |
| **“US snus”** |  |  |  |  |  |  |  |  |
| Camel Frost | <23.6 | <0.6 | <11.4 | <2.8 | <0.9 | <0.08 | <1.7 | <0.10 |
| Camel Mellow | <23.6 | <0.5 | <11.4 | <2.8 | <0.9 | <0.08 | <1.7 | <0.10 |
| Marlboro Mild | <23.6 | <0.3 | <17.1 | <4.3 | <1.2 | <0.10 | <2.2 | <0.12 |
| Marlboro Peppermint | <23.6 | <0.6 | <11.4 | <2.8 | <0.9 | <0.06 | <1.5 | <0.10 |
| Marlboro Rich | <23.6 | <0.3 | <11.4 | <2.8 | <0.9 | <0.06 | <1.5 | <0.10 |
| Marlboro Spearmint | <23.6 | <1.1 | <17.1 | <4.3 | <1.2 | <0.10 | <2.2 | <0.12 |

^a^ samples not available and therefore not analysed
